# An OmpW-dependent T4-like phage infects *Serratia* sp. ATCC 39006

**DOI:** 10.1099/mgen.0.000968

**Published:** 2023-03-30

**Authors:** Marina Mahler, Lucia M. Malone, Daan F. van den Berg, Leah M. Smith, Stan J. J. Brouns, Peter C. Fineran

**Affiliations:** ^1^​ Department of Microbiology and Immunology, University of Otago, Dunedin, New Zealand; ^2^​ Genetics Otago, University of Otago, Dunedin, New Zealand; ^3^​ Department of Bionanoscience, Delft University of Technology, Delft, Netherlands; ^4^​ Kavli Institute of Nanoscience, Delft, Netherlands; ^5^​ Bioprotection Aotearoa, University of Otago, Dunedin, New Zealand

**Keywords:** T4-like, *Winklervirus*, *Serratia *sp. ATCC39006 phage, phage genome

## Abstract

*
Serratia* sp. ATCC 39006 is a Gram-negative bacterium that has been used to study the function of phage defences, such as CRISPR–Cas, and phage counter-defence mechanisms. To expand our phage collection to study the phage–host interaction with *Serratia* sp. ATCC 39006, we isolated the T4-like myovirus LC53 in Ōtepoti Dunedin, Aotearoa New Zealand. Morphological, phenotypic and genomic characterization revealed that LC53 is virulent and similar to other *Serratia *, *Erwinia* and *Kosakonia* phages belonging to the genus *Winklervirus*. Using a transposon mutant library, we identified the host *ompW* gene as essential for phage infection, suggesting that it encodes the phage receptor. The genome of LC53 encodes all the characteristic T4-like core proteins involved in phage DNA replication and generation of viral particles. Furthermore, our bioinformatic analysis suggests that the transcriptional organization of LC53 is similar to that of *Escherichia coli* phage T4. Importantly, LC53 encodes 18 tRNAs, which likely compensate for differences in GC content between phage and host genomes. Overall, this study describes a newly isolated phage infecting *Serratia* sp. ATCC 39006 that expands the diversity of phages available to study phage–host interactions.

## Data Summary

Raw sequence reads of the phage genome are available in the National Center for Biotechnology Information (NCBI) Sequence Read Archive (SRA) database under accession number PRJNA746079. The complete genome sequence is available under NCBI GenBank accession number OP617331.1 (* Serratia* phage vB_SspM_LC53). Supplementary tables and figures are included with the online version of this article.

**Impact Statement**

The fundamental research and characterization of newly isolated phages is important to broaden our understanding of phage biology. Here, we describe the isolation of a *Serratia* T4-like phage from the genus *Winklervirus*, which highlights the global distribution of this genus. Comparison of closely related phages allows the identification of both conserved genomic features and those that change rapidly during phage evolution. The investigation of phages with highly similar genomes and with different phenotypic properties will help identify phage loci involved in host range and evasion of host resistance mechanisms. Such discoveries will increase our knowledge of phage–host interactions and improve the optimization of phages in various applications.

## Introduction

Bacteriophages (phages) are prokaryotic viruses that have been found in every environment alongside their bacterial hosts. Phages are highly diverse and employ varied infection strategies to take over and replicate within their hosts [1, 2]. In response to phages, bacteria harbour an arsenal of immune systems and can devote up to 10 % of their genome to defences [3]. The best known of these are the CRISPR–Cas (clustered regularly interspaced short palindromic repeats/CRISPR associated) and restriction–modification systems. In recent years, the discovery of additional bacterial defence systems [4–7] has deepened our understanding of the mechanisms of antiphage defences. However, most current knowledge of phage biology is biased to the study of a few model organisms and their phages (e.g. *Escherichia coli* and *Pseudomonas aeruginosa *) and focused on pathogenic bacteria. Therefore, isolating novel phages on diverse bacteria and characterizing their infection strategy is paramount to broaden our current understanding of phage–host interactions.

*Serratia* sp. ATCC 39006 (hereafter *Serratia* sp. 39006) is a flagellated enterobacterium that forms gas vesicles and was initially isolated from channel water in Cheesequake, New Jersey, USA [8]. A recent phylogenetic study has shown that *Serratia* sp. 39006 is more closely related to the genus *Dickeya* than the genus *Serratia* [9]. The authors proposed to reclassify *Serratia* sp. 39006 as *Prodigiosinella confusarubida*, and this strain is the sole representative of this genus [9]. For consistency with the existing literature, we will retain the ATCC nomenclature here. *Serratia* sp. 39006 has been studied as a model organism for the biosynthesis of secondary metabolites and antibiotics [10, 11]. Furthermore, *Serratia* sp. 39 006 harbours multiple innate anti-phage defence systems that include at least 1 restriction–modification system, 32 toxin–antitoxin systems [12] and 3 adaptive CRISPR–Cas systems (type I–E, I–F and III–A) [13]. Importantly, several *Serratia* sp. 39 006 phages belonging to diverse genera have been reported and used to examine their interactions with the host and phage defences [14–18]. Therefore, *Serratia* sp. 39 006 is a useful organism to study phage–host interactions, in particular defence and phage counter-defence strategies.

Here, to expand our phage panel to examine phage–host interactions, we isolated and characterized a newly discovered phage infecting *Serratia* sp. 39 006. Morphological and genomic analysis revealed that the phage belongs to the T4-like phages and is similar to other *Serratia* sp. 39 006, *Kosakonia* and *Erwinia* phages of the genus *Winklervirus* (*Caudoviricetes*; *Straboviridae*; *Tevenvirinae*; *Winklervirus*) isolated from diverse environments. Through genome comparison, we identified a set of conserved genes and novel features among the globally distributed phages of the genus *Winklervirus*. A detailed understanding of phage genomic features and a growing collection of phages of different levels of genomic similarity are paramount to understand their role as modulators of bacterial populations.

## Methods

### Culture conditions

Cultures of *Serratia* sp. 39 006 and *E. coli* ST18 were grown in lysogeny broth (LB) at 30 and 37 °C, respectively, in shaking conditions (160 r.p.m.). When grown on solid medium, strains were streaked onto LB agar (LBA, 1.5 % w/v) and incubated at an appropriate temperature until colony formation was observed. If required, antibiotics and supplements were added to the LB and LBA: tetracycline (Tc; 10 µg ml^−1^), kanamycin (Km; 50 µg ml^−1^), 5′aminolevulinic acid (ALA; 50 µg ml^−1^).

### Strains, primers and plasmids

Strains, primers and plasmids used in this study are listed in Tables S1, S2 and S3 and are available with the online version of this article.

### Phage isolation

Phage LC53 was isolated from wastewater samples collected from the Tahuna Wastewater Treatment Plant in Dunedin, New Zealand (45°54′16.1″S; 170°31′16.8″E). Enrichment for *Serratia* sp. 39 006 phages was performed by inoculating a *Serratia* sp. 39 006 culture with 100 µl of wastewater sample followed by incubation overnight at 30 °C with shaking (160 r.p.m.). The next day, a 10-fold dilution series of the infected culture was plated onto LB top agar overlays (0.35 % w/v) containing 100 µl of *Serratia* sp. 39 006 overnight culture. Plaques with different morphologies were picked with sterile toothpicks and used to infect new *Serratia* sp. 39 006 cultures (50 µl of *Serratia* sp. 39 006 overnight culture in 5 ml of LB media). Infected cultures were incubated overnight at 30 °C with shaking (160 r.p.m.) to allow phage propagation. Next day, cell debris was removed by centrifugation (13 000 ***g*** for 1 min) and 10-fold dilutions of the phage lysate were spotted onto LB top agar overlays containing 100 µl of *Serratia* sp. 39 006 overnight culture as explained previously. This step was repeated until plaques of homogenous appearance were obtained to ensure phage purity and purified phage stocks were stored at 4 °C.

### Phage lysate preparation

To prepare phage stocks, a double agar overlay method or propagation of the phage on a liquid culture was employed. Briefly, for the double agar overlay method, a 4 ml aliquot of LB top agar overlays (0.35 % w/v) containing 100 µl of bacterial overnight culture and 100 µl of phage stock (at sufficient concentration to produce almost confluent lysis) was poured onto LBA plates and incubated overnight at 30 °C. Overlays were harvested and pooled into a collection tube, a few drops of chloroform (NaHCO_3_-saturated) were added, and the sample was vortexed to lyse the cells. Finally, cellular debris was removed by centrifugation (2000 ***g*** for 20 min) and the new phage stock was transferred into a fresh tube. For the liquid propagation, 50 ml of LB were inoculated with 100 µl of *Serratia* sp. 39 006 overnight culture and grown at 30 °C to an optical density (600 nm) of approximately 0.2. Ten microlitres of phage stock were added and the culture was incubated at 30 °C overnight. Newly produced phages were harvested by centrifugation (2 000 ***g*** for 30 min) and filtration (0.22 µm pore size) of the supernatant. All phage stocks were stored at 4 °C.

To calculate the titre of the phage stocks, serial 10-fold dilutions of the phages were spotted (5 µl) onto an agar overlay previously seeded with 100 µl of *Serratia* sp. 39 006 overnight culture. Plates were incubated overnight at 30 °C, after which plaques were counted and the phage titre was calculated as plaque-forming units (p.f.u.) ml^−1^.

### Electron microscopy

To observe phage particles by transmission electron microscopy (TEM), 10 µl of high-titre phage stock (~10^9^ p.f.u. ml^−1^) were loaded onto plasma-glowed carbon-coated 300 mesh copper grids. After 60 s, the excess specimen was removed by blotting and 10 µl of 1 % (w/v) phosphotungstic acid (PTA) (pH 7.2) were applied to the grid to stain the samples and blotted off immediately. The grids were analysed in the Otago Micro and Nano Imaging (OMNI) facility and viewed in a Philips CM100 BioTWIN transmission electron microscope (Philips/FEI Corporation, Eindhoven, Netherlands), and images were captured using a MegaView lll digital camera (Soft Imaging System GmbH, Münster, Germany).

### Phage infection time courses

Overnight cultures of *Serratia* sp. 39 006 were diluted to an optical density (600 nm) of 0.1. The bacterial suspensions were distributed into wells of a 96-well microtitre plate and phages were added at multiplicities of infection (m.o.i.) of 0.01, 0.05, 0.1, 0.5 and 1. LB was added instead of phages for the no-phage control. The microtitre plates were incubated in a plate reader (VICTOR Nivo Multimode Microplate Reader) for 24 h at 30 °C with shaking and optical density measurements at 600 nm were taken every 10 min.

### One-step growth curve

*Serratia* sp. 39 006 cultures were grown to an optical density (600 nm) of 0.4. Phages were added at an m.o.i. of 0.01 and allowed to adsorb for 10 min at 30 °C in shaking conditions. The cultures were centrifuged at 4 000 r.p.m. for 5 min and the cell pellets with adsorbed phages were resuspended in the initial volume of LB medium. Cultures were incubated at 30 °C with shaking and samples were taken at 5 min intervals for the first 30 min and at 10 min intervals from 30 min to the end of the experiment (90 min). The concentration of free phages was determined by 10-fold serially diluting the samples and spotting on LB top agar overlay seeded with *Serratia* sp. 39 006 overnight culture. Burst size was calculated by dividing the number of phages after the burst by the number of phages at the beginning of phage maturation (*t*=0 min) after 15 min of adsorption (10 min of incubation and 5 min of centrifugation).

### Identification of the potential phage receptor

To identify the potential receptor of the phage in *Serratia* sp. 39 006 we performed transposon mutagenesis. Overnight cultures of donor (* E. coli* ST18 pKRCPN2 carrying transposon Tn-DS1028*uidA*Km [19]) and recipient (* Serratia* sp. 39006) strains were adjusted to an optical density (600 nm) of 1, washed free from antibiotics and mixed in equal ratios. The samples were spotted (20 µl) onto LBA + ALA and incubated overnight at 30 °C to allow conjugation. Next, the spots were scraped and resuspended in LB + Km to select for the transposon insertion events. The mutant pool was grown overnight and seeded onto an LBA overlay. High-titre LC53 stocks (~10^9^ p.f.u. ml^−1^) were spotted (20 µl) onto the lawn and plates were incubated at 30 °C until colonies appeared within the phage spot. Phage-resistant mutants were restreaked onto a new plate to ensure purity.

Transposon insertion sites were identified by arbitrary PCR [11]. A first round of random colony PCR was performed on phage-resistant clones using random primers PF106, PF107 and PF108 and transposon nested primers PF226 and PF1212 (Table S2). PCR products were cleaned using the GFX PCR DNA and Gel Band Purification kit (GE Healthcare) and used as template for a second round of PCR with adapter primer PF109 (that binds to the 5′ ends of PF106, PF107 and PF108) and Tn-DS1028*uidA*Km nested primers (either PF226 or PF1212). Bands were extracted, sequenced and mapped against the *Serratia* sp. 39 006 genome to detect the transposon insertion site. Efficiency of plating (EOP) assays were performed on transposon mutants (Table S1, PCF613, PCF615, PCF617, PCF618) to test phage resistance.

### Efficiency of plating assay in *Serratia* sp. 39006 mutants

Phage infectivity was assessed by EOP assays. Serial 10-fold dilutions of high titre phage stocks (~10^10^ p.f.u. ml^−1^) were spotted onto LBA overlays seeded with the appropriate *Serratia* sp. 39 006 strain (100 µl) and incubated at 30 °C overnight. The EOP was calculated as the ratio between the p.f.u. ml^−1^ in *Serratia* sp. 39 006 mutant strains and the wild-type control.

### Complementation of *Serratia* sp. 39006 transposon mutants

To complement the *ompW* transposon mutant, the vector pPF3592 was generated by Gibson assembly of the amplified *Serratia* sp. 39 006 *ompW* gene (primers PF7279 and PF7280) and the amplified pQE80L-oriT vector backbone (primers PF7281 and PF7282). Assembled pPF3592 and empty vector pQE80L-oriT were transformed into *E. coli* ST18 and the correct sequence of the *ompW* insert on pPF3592 was confirmed by Sanger sequencing. Plasmids were subsequently conjugated into *Serratia* sp. 39 006 wild-type and *ompW* transposon mutant (PCF613). Recovery of phage infectivity on the complemented transposon mutant was tested in an EOP assay by inducing the expression of *ompW* with 0.1 mM IPTG.

### Phage adsorption assay

*Serratia* sp. 39 006 overnight cultures (wild-type PCF396 or *ompW* mutant PCF613) were subcultured to an optical density (600 nm) of 0.05 and grown until they reached an optical density (600 nm) of 0.3. Cells were infected with phage LC53 at an m.o.i. of 0.01. Samples were removed at the following time points: 0 min (pre-infection) and 5, 10, 15, 20, 30 and 40 min post-infection. At each time point, samples of 200 µl of culture were taken and centrifuged for 1 min at 13 000 ***g*** to separate cells and unadsorbed phages. The supernatant (100 µl) containing phages was 10-fold serially diluted in phage buffer and spotted onto LB top agar overlays seeded with the wild-type (PCF396) strain. The plates were incubated overnight at 30 °C, and plaques were counted to calculate the number of unadsorbed phages (p.f.u. ml^−1^). A no-cell control was also included. All conditions were repeated in triplicate.

### Bacteriophage host range

Tenfold serial dilutions of high-titre phage stocks (~10^10^ p.f.u. ml^−1^) were spotted on top agar overlay seeded with overnight cultures of *Serratia* sp. 39 006, *Serratia marcescens* (*Sma*) strains 2170D, ATCC 274, SmaS6 and Sma006, *Pectobacterium atrosepticum* (*Pat*) strains SCRI1039 and SCRI1043, *Dickeya chrysanthemi* (*Dch*) strains Ec-127, 9290 and 5583, *Pectobacterium carotovorum* (*Pca*) strains RC5297, Ec-9, Ec-25, Ec-124, Ec-181 and 8974, *Pectobacterium wasabiae* (*Pwa*) 9121, *Pectobacterium oderifera* (*Pod*) 11 533, *Pantoea agglomerans* (*Pag*) strains P10c and Eh1087 and *Pseudomonas fluorescens* (*Pfl*) A506. Plates were incubated overnight at 25 °C. Plaques were analysed to distinguish successful infection (individual plaques visible) and lysis from without (visible lysis but no individual plaques). EOP was calculated as the ratio between the phage titre in tested strains and the phage titre in the isolation host *Serratia* sp. 39 006.

### Genome sequencing, annotation and comparative genomics

Phage genomic DNA (gDNA) was extracted from a high-titre phage stock (~10^9^ p.f.u. ml^−1^) using the cetyltrimethylammonium bromide (CTAB) method described elsewhere [20]. Samples were cleaned using the DNeasy Blood and Tissue kit (QIAGEN) following the manufacturer’s instructions and DNA concentration was determined by fluorimetry using the Qubit dsDNA HS Assay kit and the Qubit Fluorometer (2.0) (Invitrogen) following the manufacturer’s instructions. Phage gDNA was sent to the Massey Genome Service (MGS, New Zealand), where libraries were prepared using the Nextera XT DNA Library Preparation kit (Illumina), QC was checked with the Quant-iT dsDNA HS Assay for quantification and analysed using SolexaQA++, fastQC and fastQscreen. Sequencing was performed using Illumina MiSeq (paired-end 150 bp) and the resulting reads were processed and trimmed using SolexaQA++ (v3.1.7.1). Genome assembly was performed using SPAdes 3.9 (-spades.py, using default settings) [21] and potential coding sequences (CDSs) were identified with RASTtk (2.0) [22]. Putative functions were assigned to the CDS if multiple significant hits (e-value <10^−5^) were found by blastp against a non-redundant protein sequence database (v2.10.0) [23], Hmmer (v3.3.2) [24] or HHpred online service [25]. Domains identified by Hmmer or HHpred were added as notes to the annotation. tRNAs were identified with tRNAscan-SE (v2.0) [26] and tmRNAs were identified with aragorn [27]. The phage genome was visualized schematically using the CPT Galaxy Linear Genome Plot tool (available at https://cpt.tamu.edu/galaxy-pub) [28].

### Codon analysis

Codon usage tables for phage and host CDSs were generated by cusp (available at https://www.bioinformatics.nl/cgi-bin/emboss/cusp). In addition, the vhcub R package was used to calculate the relative codon deoptimization index (RCDI), relative synonymous codon usage (RSCU), similarity index (SiD), codon adaptation index (CAI) and GC content, including GC1, GC2 and GC3 (https://cran.r-project.org/web/packages/vhcub/index.html).

### Phylogenetic analysis

A phylogenetic tree of T4-like phages was generated with the VICTOR web service [29], a method for the genome-based phylogeny and classification of prokaryotic viruses, and modified with FigTree (v1.4.4) (http://tree.bio.ed.ac.uk/software/figtree/). Nucleotide-based intergenomic similarities were calculated with VIRIDIC [30]. To calculate the protein identities for a selected panel of phages corecruncher (https://github.com/lbobay/CoreCruncher) was used to identify the genes that all the phages encode (pident=30, query coverage=80). Core genes were concatenated for each phage and protein identities were calculated using blastp [23]. Pairwise genome comparisons were performed and visualized with clinker (v0.0.24) [31]. Variable genes were identified with the use of mmseqs2-search (default settings) [32].

## Results

### LC53 is a myovirus that infects *Serratia* sp. ATCC 39006

**Fig. 1.**
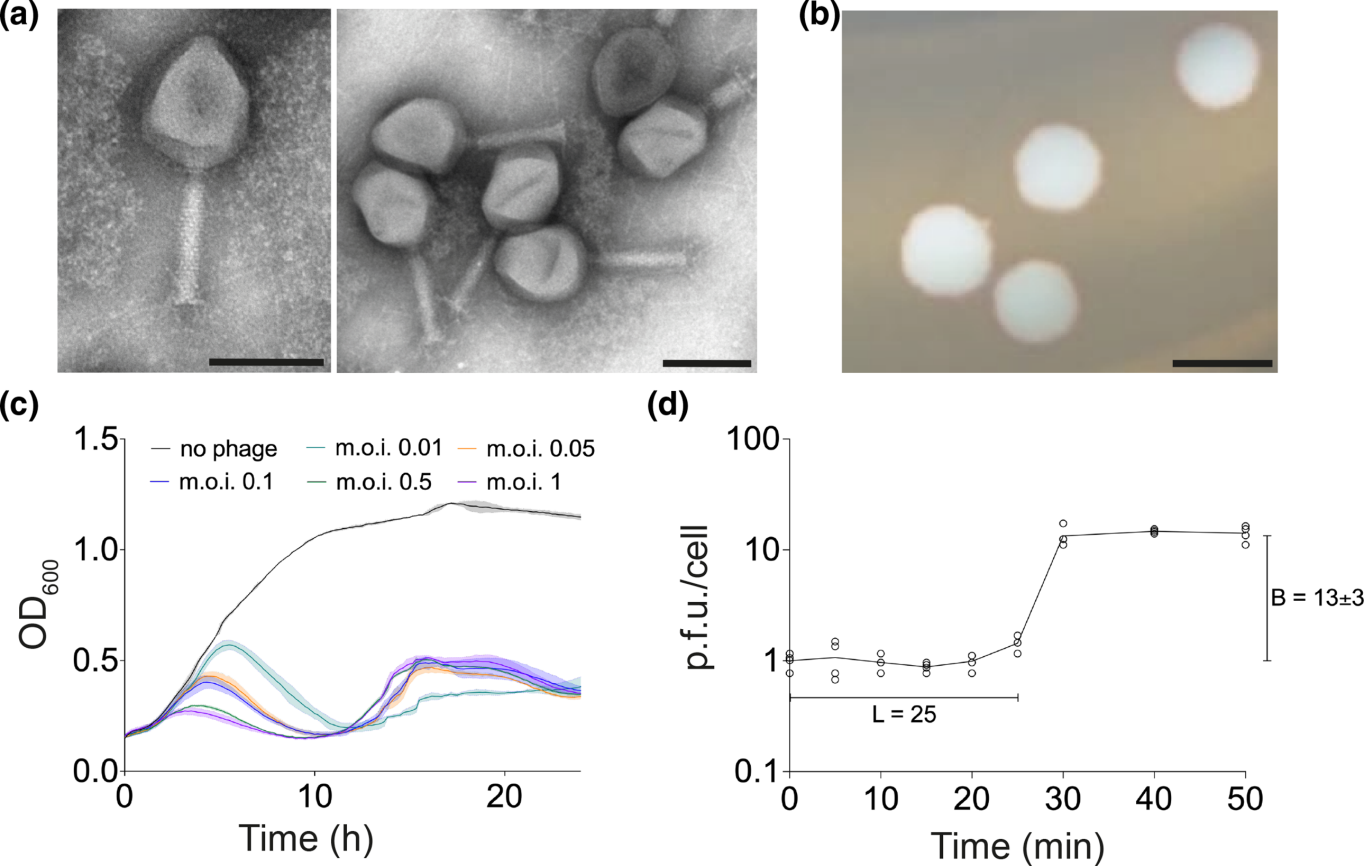
LC53 is a myovirus and rapidly lyses *Serratia* sp. 39 006. (a) Transmission electron micrographs of LC53 (scale bars, 100 nm) negatively stained. (b) Plaque morphology when infecting LB top agar overlays seeded with *Serratia* sp. 39 006 (scale bar, 2 mm). (c) Effect of the phage on culture growth of *Serratia* sp. 39 006, measured as optical density at 600 nm. Different colours indicate bacterial growth in the absence of phages or infected with phage at multiplicity of infection (m.o.i.) of 0.01, 0.05, 0.1, 0.5 or 1. Lines and shading represent the average and standard deviation of three replicates, respectively. (d) One-step growth curve of LC53 infecting *Serratia* sp. 39 006 at m.o.i. 0.01 with latency period (L) in minutes after 15 min of adsorption and burst size (B) in p.f.u. per cell. Experiments were performed in quadruplicates, and the average (line) and individual measurements are shown.

To identify new phages infecting *Serratia* sp. 39 006, we isolated phage LC53 from wastewater samples collected in Dunedin, New Zealand. TEM of the phage by negative staining showed a prolate capsid and a contractile tail (Fig. 1a), and revealed that LC53 belongs to the myovirus morphotype, which comprises tailed double-stranded DNA phages with contractile tails. When infecting a lawn of *Serratia* sp. 39 006 on 0.35 % (w/v) top agar overlays, LC53 produced clear plaques of approximately 1.5 mm in diameter with clear borders and no translucent halo surrounding the plaque (Fig. 1b). The clear plaque morphology suggests that LC53 is a virulent phage.

Observations of the infection time course in a killing assay revealed that LC53 is efficient at infecting its host, resulting in complete collapse of a liquid *Serratia* sp. 39 006 culture within the first 6 h of growth even at a low starting m.o.i. of 0.01 (Fig. 1c). This infection pattern suggests a rapid lytic life cycle. To further investigate the infection cycle, we performed a one-step growth curve and found that the phage has a latency period of 25 min after 15 min of adsorption and a burst size of approximately 13 phages per infected cell (Fig. 1d). These values are similar to those reported for the related *Serratia* sp. 39 006 phage CBH8 [18]. In summary, LC53 is a myovirus infecting *Serratia* sp. 39 006 and our data suggest that it is a virulent phage in this host.

### LC53 requires OmpW for successful infection

**Fig. 2.**
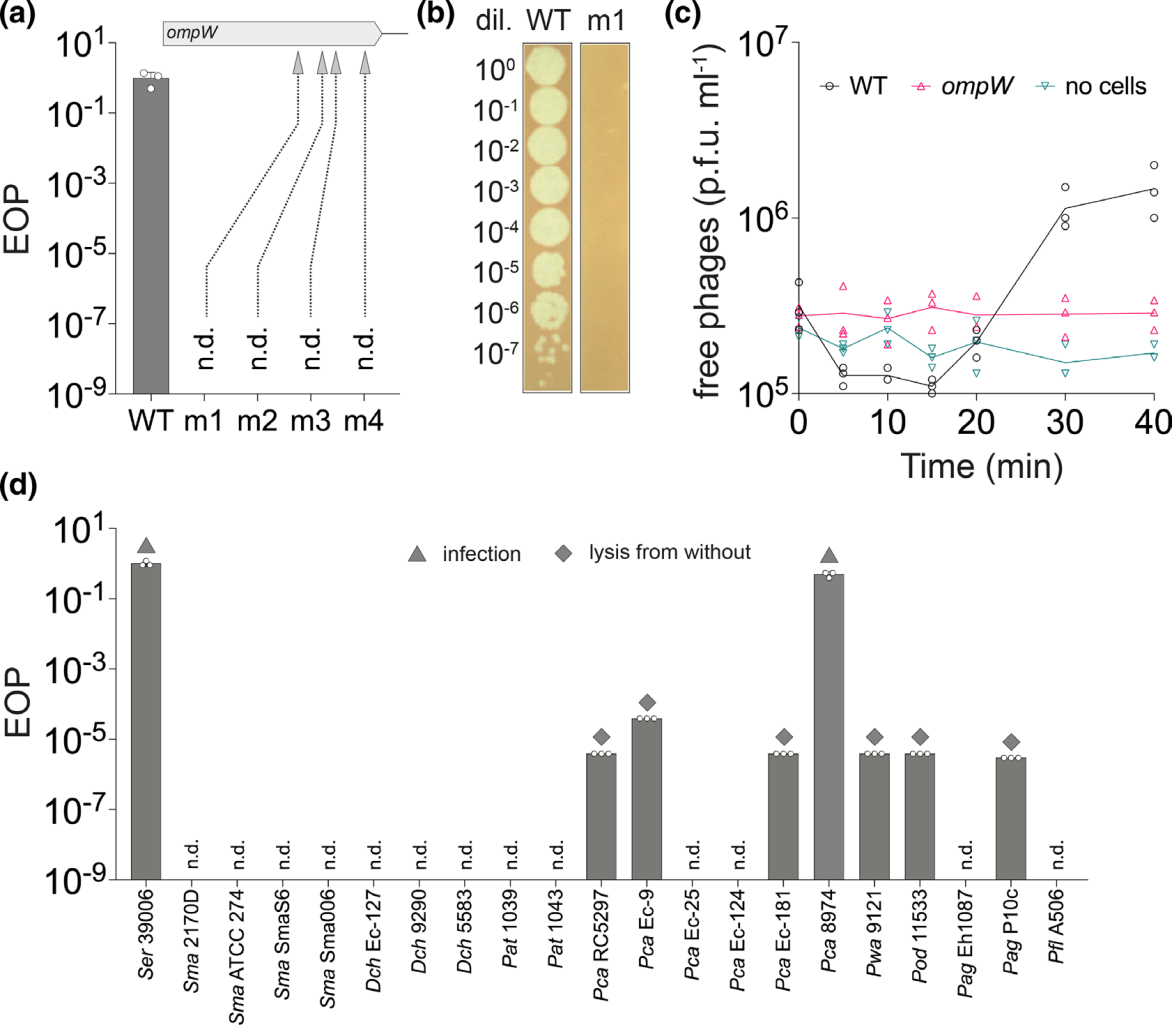
Mutations in *ompW* cause LC53 phage resistance and LC53 also infects some *Pectobacterium* strains. (a) EOP assay on receptor mutant *Serratia* sp. 39 006 stains m1 (PCF613), m2 (PCF615), m3 (PCF617) and m4 (PCF618) with different transposon insertion sites within the *ompW* gene. Bars represent the average of three replicates and error bars show the standard deviation. None detected (n.d.) was noted when no plaque formation was observed. (b) EOP assay and LC53 plaque morphology on LBA overlays seeded with *Serratia* sp. 39 006 WT and receptor mutant (**m1**). (c) Adsorption assay of LC53 with *Serratia* sp. 39 006 WT and an *ompW* mutant at m.o.i. 0.01 shown as change in free phages in the media. Phage burst is visible from approximately 20 min. A negative control with no cells was included. Lines represent the average of three replicates. (d) EOP assay on isolation host *Serratia* (*Ser*) sp. 39 006, *Serratia marcescens* (*Sma*) strains 2170D, ATCC 274, SmaS6 and Sma006, *Dickeya chrysanthemi* (*Dch*) strains Ec-127, 9290 and 5583, *Pectobacterium atrosepticum* (*Pat*) strains 1039 and 1043, *Pectobacterium carotovorum* (*Pca*) strains RC5297, Ec-9, Ec-25, Ec-124, Ec-181 and 8974, *Pectobacterium wasabiae* (*Pwa*) 9121, *Pectobacterium odorifera* (*Pod*) 11 533, *Pantoea agglomerans* (*Pag*) strains Eh1087 and P10c, and *Pseudomonas fluorescens* (*Pfl*) A506. Successful infection visible as individual plaques is indicated with a triangle. Interaction of the phage with the strain that resulted in clearing of the bacterial lawn, but no formation of individual plaques (lysis from without), is indicated with a diamond. The EOP for lysis from without was calculated by counting one p.f.u. for the first dilution step that did not result in clearing of the bacterial lawn. Bars represent the average of three replicates and error bars show the standard deviation.

To identify the potential cell surface receptor required by LC53 for successful adsorption, a *Serratia* sp. 39 006 transposon mutant pool was challenged with LC53. Phage-resistant colonies were isolated and further examined. Four phage-resistant mutants showed complete inhibition of plaque formation (Figs 2a, b). Sequencing revealed that these resistant mutants had transposon insertions in unique positions within the *ompW* gene (Fig. 2a). OmpW is an outer-membrane eight-stranded β-barrel porin predicted to transport small hydrophobic compounds [33] and is known to serve as a colicin S4 receptor in *E. coli* [34]. As plaque formation was completely abolished in all the tested *ompW* mutants (m1, m2, m3 and m4), and no escape phages were observed, we conclude that OmpW is crucial for infection by LC53 and is likely to be the primary receptor. The influence of any potential point mutants on the observed phenotype could be excluded by restoring the phage infectivity through complementation of the *ompW* transposon mutant (m1, Fig. S1). To directly test the effect of OmpW on LC53 binding to *Serratia* sp. 39 006, adsorption assays were performed. Phage adsorption to the wild-type strain was observed as a decrease in the titre of free phages during the first 10 to 15 min, followed by an increase in free phages after the burst (Fig. 2c). In contrast, no change in free phage abundance was observed with the *ompW* mutant, indicating that LC53 cannot adsorb to this strain. A similar profile for the number of free phages was detected for a negative control lacking any host cells. These data demonstrate that LC53 requires OmpW for successful adsorption.

### LC53 has a narrow host range

Since OmpW is a widely conserved outer-membrane protein, we were interested in testing the ability of LC53 to infect other strains. The isolation host, *Serratia* sp. 39 006, was recently proposed to be reclassified as the only member of the new genus *Prodigiosinella* (i.e. *Prodigiosinella confusarubida*) [9]. This new genus is closely related to the genera *Dickeya *, *Pectobacterium* and *Serratia *, but represents a distinct lineage [9]. Therefore, we tested whether several related *Dickeya *, *Pectobacterium* and *Serratia* strains, as well as more distantly related *Pantoea* and *Pseudomonas* strains, were infected. The host range of LC53 was relatively narrow, with successful infection limited to the isolation strain *Serratia* sp. 39 006 and *P. carotovorum* 8974 (Fig. 2d). In addition, LC53 caused lysis from without on some strains (Fig. S2), which was visible as clearance of the bacterial lawn without individual plaques at high phage titres [35]. This lysis-from-without phenotype was observed for *P. carotovorum* strains RC5297, Ec-9 and Ec-181, *P. wasabiae* 9121, *Pectobacterium odorifera* 11 533 and *P. agglomerans* P10c, and suggests that these strains contain the LC53 receptor but infection may be limited by other factors, such as defence systems or genetic/metabolic requirements. In summary, LC53 requires the presence of OmpW for infection and can infect some strains from different genera, including *Serratia* sp. 39 006 and one *P. carotovorum* strain.

### Phage LC53 has a lower GC content than its host and encodes tRNAs

**Fig. 3.**
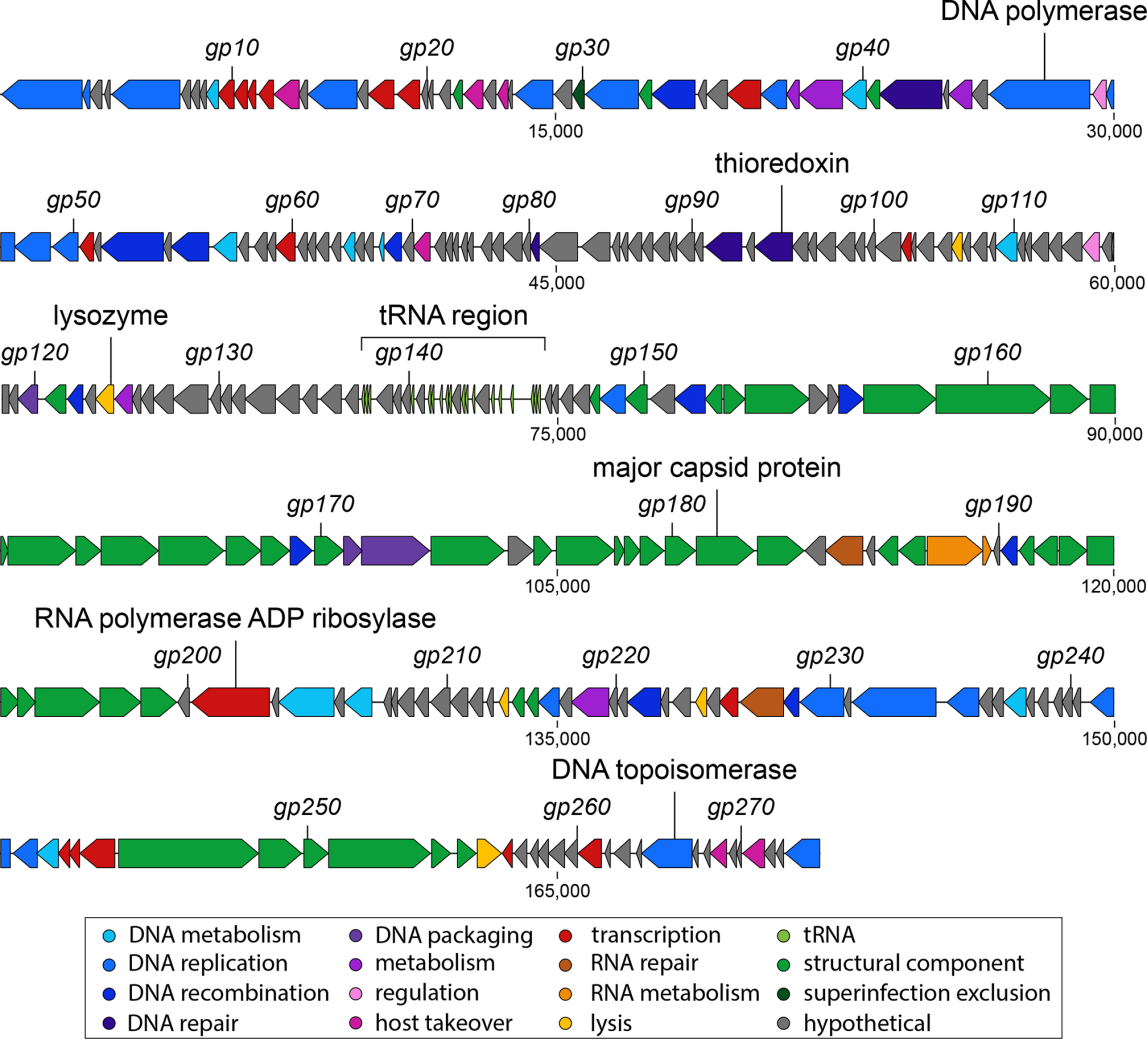
Linear genome map of phage LC53. Arrows represent identified CDSs and are coloured according to predicted functions, as shown in the key. The tRNA cluster is indicated by a bracket and shown in more detail in Fig. 4a. Refer to Table S4 for the full annotation of genes.

To characterize LC53 and its relationship to other phages in more detail, we sequenced, assembled and annotated its genome. LC53 has a circularly permuted dsDNA genome of 172 075 bp with a GC content of 38.7 %, which is approximately 8 % lower than the GC content of *Serratia* sp. 39 006 (47 %). We identified 274 CDSs in the phage genome, of which half (136) were classified as hypothetical proteins of unknown function (Fig. 3). The remaining (138) CDSs could be assigned a putative function based on homology to annotated phage proteins. Most of those annotated CDSs could be classified into different functional groups (Fig. 3), with DNA-related functions (49 CDS) and structural components (47 CDS) being the most abundant. Further CDSs were classified to have transcription and RNA related functions, metabolic or regulatory functions, or functions involved in host takeover and lysis. In agreement with our phenotypic observations, we did not identify any genes (such as integrases) that are characteristic for temperate phages, suggesting that LC53 is a virulent phage.

**Fig. 4.**
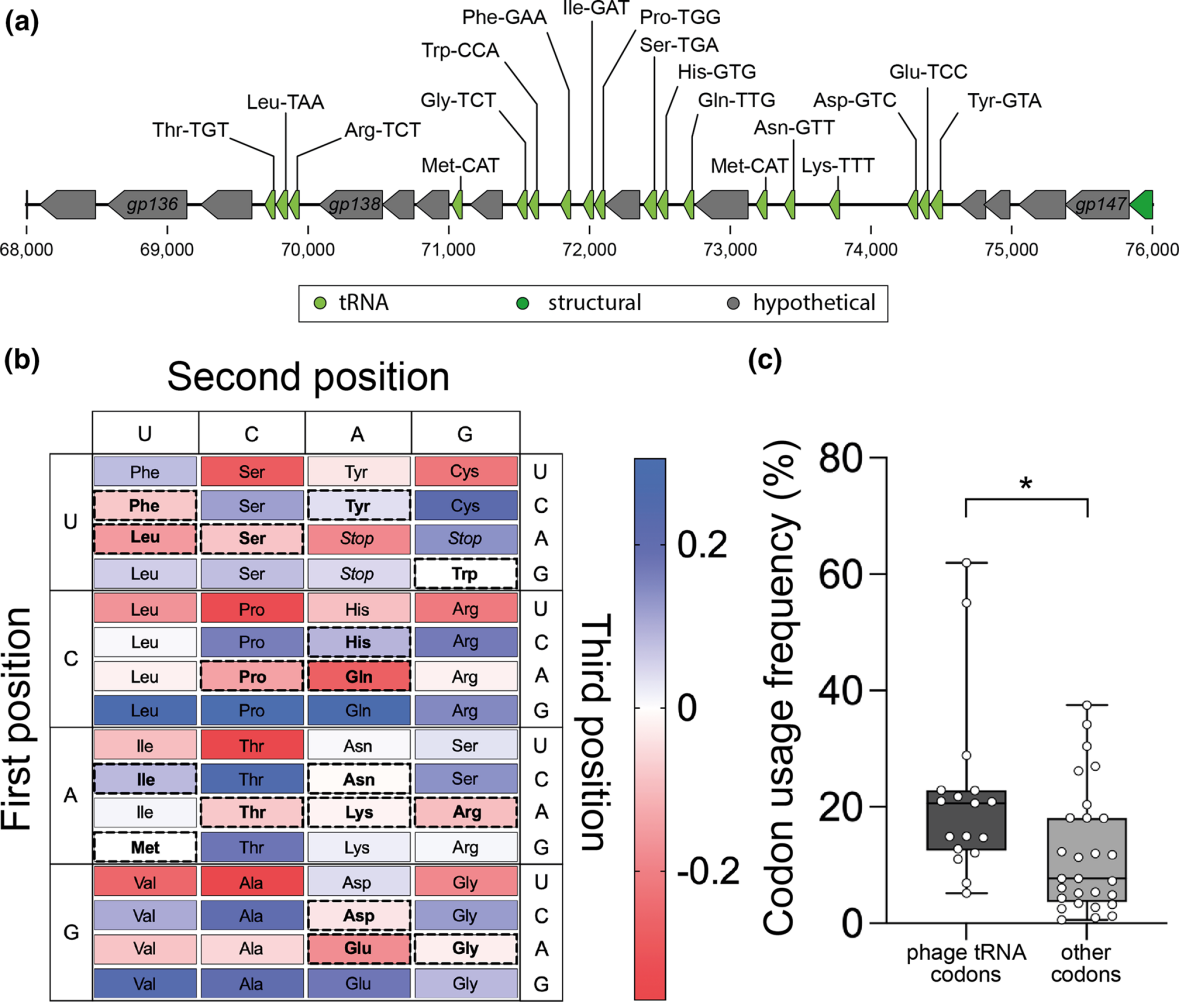
tRNAs encoded by LC53 favour low-GC-content anticodons. (a) Map of the genomic locus (highlighted in Fig. 3) that encodes the 18 tRNAs found in LC53. (b) Difference in codon preference between host and phage. Blue codons are more frequently used by the host and red codons are more frequently used by the phage. Codons for which the phage carries the appropriate tRNA are marked with dashed boxes. (c) Phage codon usage frequencies for codons recognized by phage-encoded tRNAs compared to all the other codons that are only recognized by the host-encoded tRNAs. Data is represented as boxes from the 25th to 75th percentiles with whiskers representing the minimum and maximum values. *P<0.05 (Welch two sample *t*-test, *t*=2.2637, df=25.62, *P*=0.032).

Additionally, we identified 18 tRNAs within the genome of LC53 (Figs 3 and 4a). Phage tRNAs have been hypothesized to benefit phage replication [36, 37]. To test this hypothesis in LC53, we first analysed if the presence of tRNAs in the phage genome correlates with increased usage of the corresponding codons by generating a codon usage table for the phage and *Serratia* sp. 39 006 host (Fig. 4b). Next, we compared the codon frequency of tRNAs encoded by the phage with those that are only encoded by the host (Fig. 4c). We observed that the codons of tRNAs encoded by the phage are used more frequently (mean codon frequency=21.7) than codons that are only encoded by the host tRNAs (mean codon frequency=12.2) (Welch two sample *t*-test, *t*=2.2637, df=25.62, *P*=0.032). Furthermore, the anticodons of the phage-encoded tRNAs contain a lower GC % than the host tRNA anticodons (LC53: codon GC %=38, *Serratia* sp. 39 006: codon GC %=50). Therefore, we hypothesize that these tRNAs might enable the phage to compensate for the difference in GC content compared to the host, to allow efficient translation, as observed previously [38]. In summary, LC53 is a 172 kb dsDNA phage with a lower GC content than *Serratia* sp. 39 006, and this disparity in GC content may be mitigated by encoding 18 tRNAs for low-GC-content anticodons.

### LC53 is a T4-like phage

The genome of LC53 shows genetic and organizational similarity to T4-like phages and several CDSs in LC53 were annotated as homologues of genes encoded by *Escherichia* phage T4 (Fig. 3 and Table S4). A genetic comparison between LC53 and T4 revealed that LC53 encodes all T4-like core genes [39] (Table S5). The similarity to T4-like phages suggested that the transcriptional organization of LC53 may be similar to T4 (Table S6). Transcription of early genes in T4 is initiated by strong promoters that compete for the host RNA polymerase [40] and the Alc protein that modifies residues of the host RNA polymerase to further increase transcription of phage genes by inhibition of host transcription [41, 42]. In agreement, an LC53 gene (*gp227*, UYM28880.1) encodes Alc and is annotated as an inhibitor of host transcription. Conversion from transcription of early to middle T4 promoters is controlled by two processes; downstream extension of early transcripts into middle genes and activation of middle promoters by a process known as sigma appropriation [43]. Several proteins are involved in these two processes. The AsiA and MotA proteins are responsible for the sigma appropriation and activation of middle promoters containing a MotA box [43–45]. LC53 genes *gp255* (UYM28908.1) and *gp261* (UYM28914.1) encode proteins homologous to T4 anti-sigma 70 protein AsiA (57 % aa identity) and MotA (50 % aa identity), respectively. The T4 ComC-α protein (Gp10, UYM28663.1 and Gp11, UYM28664.1 in LC53) is involved in the downstream extension of the early transcripts by increasing the stability of the synthesized RNAs [46]. Additionally, the two ADP ribosylation enzymes ModA and ModB also contribute to the conversion from early to middle transcription [47]. In LC53, a gene annotated as *modB* (*gp19*, UYM28672.1) has homology to both *modA* and *modB* with a low amino acid similarity (30.5 and 42.5 % aa identity to ModA and ModB, respectively). It has been demonstrated that ModA is not essential for the rapid decrease in early transcription during conversion to T4 middle transcription [48]. This suggests that the absence of ModA in LC53 does not result in any major changes in the conversion from early to middle transcription in comparison to the known mechanism of T4. During transcription of T4 middle genes, inhibition of host transcription is no longer required, as the host DNA is degraded by the activity of the endonuclease II and IV encoded by *denA* and *denB*, respectively [49, 50]. We predict that the *Serratia* sp. 39 006 genome is also degraded quickly after LC53 infection, as homologues of both *denA* (*gp229*, UYM28882.1) and *denB* (*gp271*, UYM28924.1) were identified. Finally, transcription of T4 late genes occurs in parallel with phage DNA replication and involves the activity of two activators, the sliding clamp protein Gp45 and RNA polymerase sigma-like factor Gp55, together with a co-activator, the RNA polymerase-associated protein Gp33 [51]. These proteins facilitate recognition of the T4 late promoters with the conserved sequence 5′-TATAAATA-3′ at the −10 site by the host RNA polymerase [52]. LC53 homologues of T4 Gp45 (LC53 Gp50, UYM28703.1 64 % aa identity), Gp55 (LC53 Gp60, UYM28713.1 76 % identity) and Gp33 (LC53 Gp245, UYM28898.1 60 % identity) were annotated as sliding clamp, sigma factor for late transcription and phage late transcription accessory protein. Furthermore, the T4 conserved sequence for late promoters was found multiple times in the LC53 genome and in most cases was located upstream of structural or other late expressed genes (Table S7).

We were also interested in the DNA packaging mechanism of LC53 and identified genes similar to those encoding the T4 small (LC53 Gp171, UYM28824.1) and large (LC53 Gp172, UYM28825.1) terminase proteins, as well as the portal protein (LC53 Gp176, UYM28829.1) that mediate the headfull DNA packaging of T4 [53] (Table S8). Overall, the similar organization and the identification of homologous genes on the LC53 genome suggest that it has a similar transcriptional organization and DNA packaging mechanism to T4.

### LC53 is closely related to other phages of the genus *Winklervirus*

**Fig. 5.**
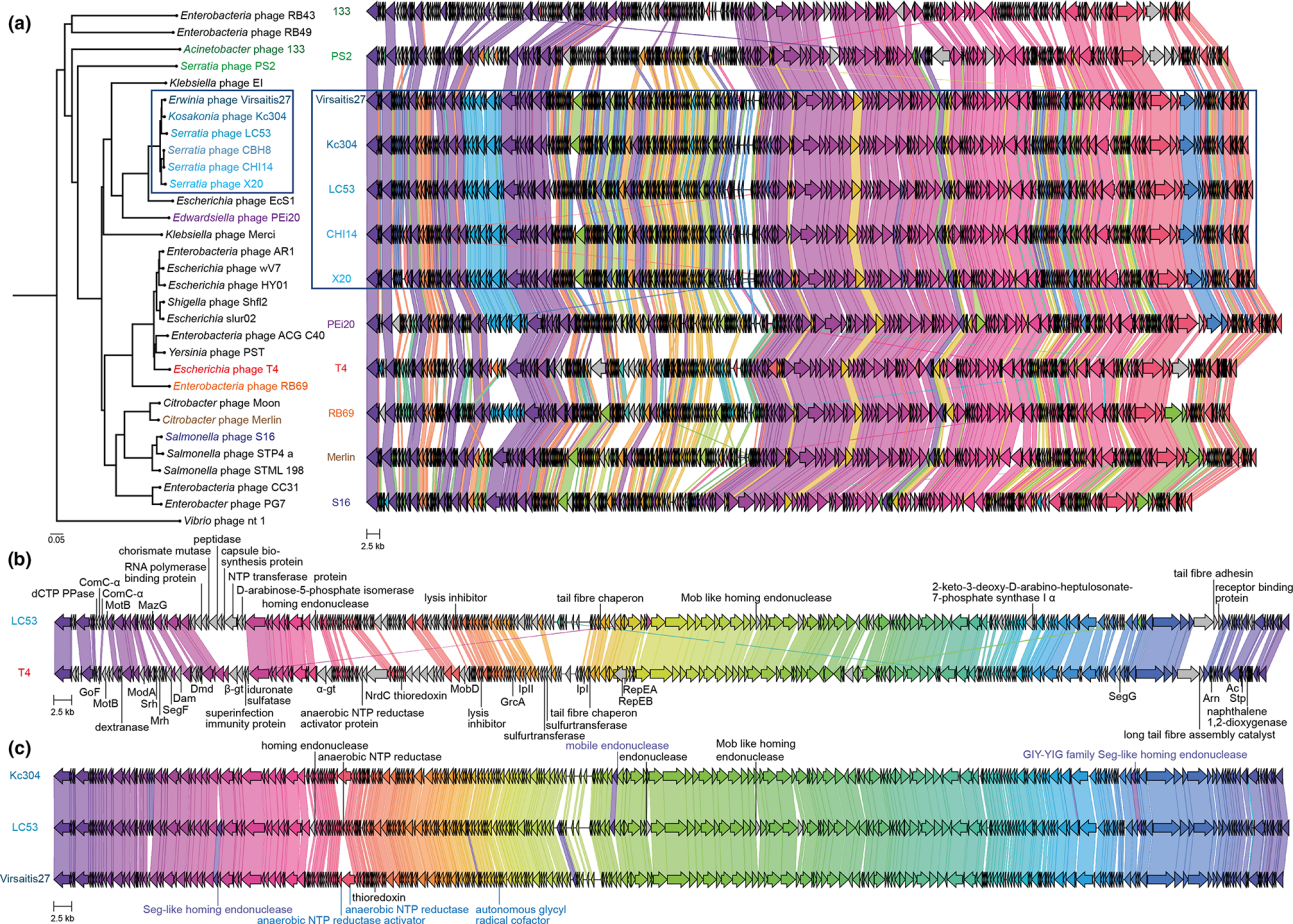
Phylogeny and genome similarity within the T4-like phage family and winklerviruses. (a) Phylogenetic tree of T4-related phages based on whole genomes and genome alignments of some selected phages. The genus *Winklervirus* is marked with a blue box. *Serratia* phage CBH8 is not represented in the genome alignments, as it is almost identical to *Serratia* phage CHI14 (19 point mutations). Branch lengths are scaled according to nucleotide substitutions per site. (b) Detailed comparison between LC53 and T4 genomes with indications of proteins for which no homologue was found. Hypothetical proteins of unknown function are not labelled. (c) Detailed genome comparison between LC53 and its closest relatives *Kosakonia* phage Kc304 and *Erwinia* phage Virsaitis27, with indication of proteins of LC53 lacking homologues in Kc304 and Virsaitis27 (top row, black font), proteins found in LC53 and either Kc304 or Virsaitis 27 (top and bottom row, respectively, purple font), proteins only found in Virsaitis27 but not in LC53 and Kc304 (bottom row, black font), and proteins found in Kc304 and Virsaitis27 without a homologue in LC53 (bottom row, blue font). Only CDSs annotated with a putative function are labelled.

A phylogenetic analysis of LC53 revealed that the phage is closely related to the *Serratia* sp. 39 006 phages CHI14, CBH8 and X20, as well as *Kosakonia* phage Kc304 and *Erwinia* phage Virsaitis27 (Fig. 5a). These phages represent the entire collection of phages currently classified within the genus *Winklervirus* (*Caudoviricetes*; *Straboviridae*; *Tevenvirinae*; *Winklervirus*) by the National Center for Biotechnology Information (NCBI). The high nucleotide-based intergenomic similarity (≥92 %) and protein identity (≥98.9 %) to all five phages and calculations using the VIRIDIC algorithm [30] classify LC53 as a new species (94.7 % DNA sequence similarity [54]) within the genus *Winklervirus*. The closest relatives outside this genus, the T4-like *Escherichia* phage EcS1 (genus *Kagamiyamavirus*) and *Edwardsiella* phage PEi20 (genus *Kanagawavirus*) only share 66 and 49% intergenomic similarity, respectively (Table S9).

To identify differences between the *Winklervirus* LC53 and the well-characterized T4 phage, a whole-genome alignment was performed and the presence and absence of CDSs investigated (Fig. 5b). For 112 CDSs encoded by LC53, no homologous CDS was found in T4 based on a cut-off of 30 % aa identity (Table S10). Most of the LC53 CDSs were assigned as hypothetical proteins of unknown function. Some differences could be explained by low sequence identity rather than absence of a homologous protein in T4, such as observed for the genes encoding the dCTP pyrophosphatase, ComC-α, MotB, RIIA, Hoc or endonuclease proteins. Overall, on the LC53 genome we found homologues of all the T4-like core genes that were defined previously [39]. The CDSs between the genes encoding UvsX and the DNA polymerase seem to be highly variable between LC53 and T4 (see Fig. 5b, genes in T4 located between those encoding T4 β-glucosyltransferase and iduronate sulfatase are missing in LC53). While there are 5 CDSs encoded between these two genes in T4, 12 CDSs were identified in the same locus for LC53 and none are homologous to the CDSs found in T4. Furthermore, homologues of most putative structural components of LC53 were found in T4, with the exception of *gp148* (UYM28801.1), *gp251* (UYM28904.1), *gp252* (UYM28905.1) and *gp253* (UYM28907.1), which are predicted to be involved in the formation of the long tail fibre, tail fibre adhesin and receptor-recognizing proteins.

Next, we investigated the differences between LC53 and its closest relatives within the genus *Winklervirus* – *Kosakonia* phage Kc304 and *Erwinia* phage Virsaitis27. LC53 shares 92.5 and 91.3 % DNA sequence identity with those phages, respectively (Fig. 5c). Seven CDSs were unique to LC53, with three annotated as hypothetical proteins, three as endonucleases (two homing endonucleases and one putative endonuclease) and one as an anaerobic NTP reductase. Two functionally related CDSs encoding an anaerobic dNTP reductase and a ribonucleotide reductase-activating protein were present in Kc304 and Virsaitis27, but no homologue was found in LC53. Interestingly, one of the putative unique endonucleases and hypothetical proteins of LC53 was homologous to a corresponding CDS in T4. Furthermore, two homing endonucleases were only found in LC53 (*gp151*, UYM28804.1 and *gp244*, UYM28897.1) and Kc304, but not Virsaitis27, while one homing endonuclease was only found in LC53 (*gp40*, UYM28693.1) and Virsaitis27, but not Kc304. This observation suggests that homing endonucleases are a common source of variation between closely related phages of the genus *Winklervirus*. In summary, LC53 is a distinct member of the genus *Winklervirus* but has high sequence similarity to other members of this genus.

## Discussion

We isolated and characterized a *Serratia* sp. 39 006 phage that is a myovirus and a close relative to other phages of the genus *Winklervirus* (family *Straboviridae*). LC53 has a lytic life cycle and is dependent on the presence of OmpW – its likely receptor – for successful infection. The host used to isolate the phage, *Serratia* sp. 39 006, was recently proposed to be reclassified as *Prodigiosinella confusarubida*, the only member of the new genus *Prodigiosinella*. This new genus is closely related to phytopathogenic bacteria of the genera *Dickeya* and *Pectobacterium *. Infection of some strains of those genera demonstrated that the inter-genera host range of LC53 was strain-specific.

Relatives of *E. coli* phage T4 that infect various bacterial hosts such as *Enterobacter *, *Aeromonas *, *Acinetobacter *, *Klebsiella *, *Vibrio *, *Pseudomonas *, and even marine bacteria such as *Synechococcus* and *Prochlorococcus,* are highly abundant and have been isolated all around the world [39, 55, 56]. LC53 and other highly similar members of the *Winklervirus* genus have been isolated from either the same strain (* Serratia* sp. 39006) or distinct genera at different time points and regions of the world (e.g. the UK, New Zealand and the Czech Republic) [18, 57]. The isolation of highly similar viruses demonstrates the global distribution of these phages. Furthermore, T4-like phages are typically highly virulent, resulting in the collapse of an infected culture and a relatively high burst size. LC53 was also found to completely arrest growth of a host culture, even at low starting m.o.i. However, the burst size of LC53 was rather low compared to T4-like phages, but was similar to that of *Serratia* sp. 39 006 phage CBH8, which had a burst size of 22 [18]. Studies with T4 have shown that burst size is highly dependent on growth characteristics of the host [58–60]. Those characteristics vary a lot between *E. coli* and *Serratia* (e.g. doubling time) and may have an effect on the replication of the phage.

T4-like phages show a high level of genetic variability but share a set of conserved genes responsible for replication, temporal expression and packaging of the viral chromosome [39, 61]. In the case of LC53, only half of the CDSs encoded could be assigned a predicted function. However, the structural components and proteins involved in DNA replication were conserved between T4 and LC53. Additionally, LC53 appears to have a similar mechanism of mediating transcription via host RNA polymerase modification to that demonstrated for T4. Phage T4 recognizes *E. coli* by either binding to the lipopolysaccharides or OmpC via the distal tip of the long tail fibre [62–64]. Genes encoding proteins involved in the formation of the long tail fibre were highly variable among the otherwise conserved structural genes between T4 and LC53. This suggests that the receptor-binding domain of LC53 that likely recognizes OmpW or related porins on the cell surface is part of the long tail fibre protein. Furthermore, the genetic diversity between the highly conserved genus *Winklervirus* was mainly explained by the presence or absence of certain homing endonucleases, a class of mobile genetic elements known to promote their spread within and between genomes [39, 65]. Together, LC53 is a winklervirus, showing the typical morphological and genetic features of T4-like phages, demonstrating the geographically widespread success of this family of bacterial viruses. The similarity to T4 allows us to predict many characteristics of LC53 from the extensive knowledge gained from investigations on T4. Those predicted characteristics can be applied to study a new phage–host system with *Serratia* sp. 39 006. Therefore, LC53 will provide a new phage model to study its interaction with *Serratia* sp. 39 006 and its phage defence systems.

## Supplementary Data

Supplementary material 1
