# Supplementary material for: An OmpW-dependent T4-like phage infects Serratia sp. ATCC 39006
Source: Microb Genom. 2023 Mar 30;9(3):mgen000968. doi: 10.1099/mgen.0.000968 (PMC10132071; doi:10.1099/mgen.0.000968)
Supplement: Supplementary material 1 [file mgen-9-968-s001.pdf]

Supplementary information

An OmpW-dependent T4-like phage infects *Serratia* sp. ATCC 39006

Marina Mahler[1,2,3#], Lucia M. Malone[1,3,4#], Daan F. van den Berg[3,4], Leah M. Smith[1,2], Stan J. J. Brouns[3,4], Peter C. Fineran[1,2,5*]

1 Department of Microbiology and Immunology, University of Otago, Dunedin, New Zealand

2 Genetics Otago, University of Otago, Dunedin, New Zealand

3 Department of Bionanoscience, Delft University of Technology, Netherlands

4 Kavli Institute of Nanoscience, Delft, Netherlands

5 Bioprotection Aotearoa, University of Otago, Dunedin, New Zealand

#These authors contributed equally

*Correspondence: peter.fineran@otago.ac.nz

Table S1: Strain list.

Species	Strain	Description	Notes	Reference
E. coli	ST18	Auxotrophic donor for biparental conjugation	Requires ALA	(1, 2)
Serratia sp. ATCC 39006	LacA	Lac- EMS mutant, denoted WT		(3)
Serratia sp. ATCC 39006	PCF396	ΔpigA-O, denoted WT		(4)
Serratia sp. ATCC 39006	PCF613 (m3)	*ompW*::Tn-DS1028*uidA*Km, LacA-derivative	KmR	This study
Serratia sp. ATCC 39006	PCF615 (m1)	*ompW*::Tn-DS1028*uidA*Km, LacA-derivative	KmR	This study
Serratia sp. ATCC 39006	PCF617 (m2)	*ompW*::Tn-DS1028*uidA*Km, LacA-derivative	KmR	This study
Serratia sp. ATCC 39006	PCF618 (m4)	*ompW*::Tn-DS1028*uidA*Km, LacA-derivative	KmR	This study
Serratia marcescens	2170D	Wild-type, extracellular nuclease producer		(5)
Serratia marcescens	ATCC 274	Wild-type ATCC reference strain		(6)
Serratia marcescens	SmaS6	Wild-type		Salmond Lab, UK
Serratia marcescens	Sma006	Wild-type		Salmond Lab, UK
Pectobacterium atrosepticum	SCRI1039	Wild-type, contains native pECA1039 plasmid with ToxIN toxin-antitoxin abortive infection system		(7, 8)
Pectobacterium atrosepticum	SCRI1043	Wild-type, Perthshire, Scotland		(9)
Pectobacterium carotovorum	RC5297	Wild-type, phage ZF40-susceptible strain		(10, 11)
Pectobacterium carotovorum	Ec-9	Wild-type, potato tubers, Cheltenham, NZ		Plant & Food Research Ltd (PFR, NZ)
Pectobacterium carotovorum	Ec-25	Wild-type, potato tubers, Matamata, NZ		PFR, NZ
Pectobacterium carotovorum	Ec-124	Wild-type, potato tubers, Matamata, NZ		PFR, NZ
Pectobacterium carotovorum	Ec-181	Wild-type, potato tubers, Pukekawa, NZ		PFR, NZ
Pectobacterium odorifera	ICMP 11533	Wild-type, witloof chicory, Loiret, France		(12)
Pectobacterium wasabiae	IMCP 9121	Wild-type, horse-radish, Nagano Prefecture, Japan		**(12)**
Pantoea agglomerans	P10c	Wild-type, Fireblight biocontrol agent		(13)
Pantoea agglomerans	Eh1087	Wild-type, Fireblight biocontrol agent		(14)
Dickeya chrysanthemi	Ec-127	Wild-type, potato tubers, Matamata, NZ		PFR, NZ
Dickeya chrysanthemi	ICMP 9290	Wild-type, kumara, Papua New Guinea		PFR, NZ
Dickeya chrysanthemi	ICMP 5583	Wild-type, tomato, Cook Islands		PFR, NZ

Table S2. Primer list.

Name	Sequence	Description
PF106	GACCACACGTCGACTAGTGCNNNNNNNNNNAGAG	Random primed PCR primer 1
PF107	GACCACACGTCGACTAGTGCNNNNNNNNNNACGCC	Random primed PCR primer 2
PF108	GACCACACGTCGACTAGTGCNNNNNNNNNNGATAC	Random primed PCR primer 2
PF109	GACCACACGTCGACTAGTGC	Random primed PCR adapter primer
PF226	CATAAGGGACTCCTCATTAAG	Nested primer
PF1212	CGGGAATTCTCATGTTTGAC	Nested primer
PF7279	GCTTAATTAGCTGAGCTTGGACTCCCCAAGAAACATGTCAGGATG	*Serratia* sp. 39006 *ompW* fwd
PF7280	GATGGAGTTCTGAGGTCATTACTGGGCCGTACAATAACTAAGCGA	*Serratia* sp. 39006 *ompW* rev
PF7281	CCAGTAATGACCTCAGAACTCC	pQE80L-oriT backbone fwd
PF7282	GGAGTCCAAGCTCAGCTAAT	pQE80L-oriT backbone rev

Table S3. Plasmid list.

Name	Features	Description	Reference
pKRCPN2	carrying transposon Tn-DS1028*uidA*Km	TcR (plasmid), KmR (transposon), RP4oriT, oriR6K	(15)
pQE80L-oriT	Plasmid for IPTG-inducible gene expression	AmpR, T5-lac, lacI, pBR322, RP4oriT	(16)
pPF3592	*Serratia* sp. 39006 *ompW* expression	AmpR, T5-lac, lacI, pBR322, RP4oriT	This study

Table S4. Putative genes of LC53.

gp	locus tag	Description	Amino acids	Domains	pfam number	Closest homologue			
						Phage	locus tag	q cov %	aa id %
1	UYM28654.1	protector form prophage-induced early lysis (rIIA)	723			Kc304	QYN80447.1	100	99.86
2	UYM28655.1	rIIA lysis inhibitor	68			Kc304	QYN80448.1	100	100
3	UYM28656.1	hypothetical protein	112			Kc304	QYN80449.1	100	100
4	UYM28657.1	hypothetical protein	51			CHI14	YP_009609329.1	100	100
5	UYM28658.1	DNA topoisomerase II large subunit	613	Histidine kinase-, DNA gyrase B-, and HSP90-like ATPase; DNA gyrase B	PF02518; PF00204	Kc304	QYN80451.1	100	99.35
6	UYM28659.1	hypothetical protein	83			Virsaitis27	UJJ21990.1	100	98.78
7	UYM28660.1	hypothetical protein	83			X20	YP_010092157.1	100	100
8	UYM28661.1	family putative regulatory protein	60			CHI14	YP_009609332.1	100	100
9	UYM28662.1	dCTP pyrophosphatase	113			CHI14	YP_009609333.1	100	100
10	UYM28663.1	comC-alpha protein	145			Kc304	QYN80456.1	100	95.14
11	UYM28664.1	comC-alpha protein	119			Virsaitis27	UJJ21995.1	100	99.15
12	UYM28665.1	modifier of suppressor tRNAs	76			Kc304	QYN80458.1	100	100
13	UYM28666.1	transcription regulatory protein motB	113			Kc304	QYN80459.1	100	100
14	UYM28667.1	exonuclease A	228	3'-5' exoribonuclease Rv2179c-like domain	PF16473	Kc304	QYN80460.1	100	99.56
15	UYM28668.1	hypothetical protein	78			X20	YP_010092165.1	100	98.7
16	UYM28669.1	DNA helicase	442	AAA domain; Dda helicase SH3 domain; UvrD-like helicase C-terminal domain	PF13604; PF18343; PF13538	CHI14	YP_009609341.1	100	99.32
17	UYM28670.1	hypothetical protein	99			X20	YP_010092167.1	100	100
18	UYM28671.1	putative anti-sigma factor	232			CHI14	YP_009609343.1	100	100
19	UYM28672.1	NAD-protein ADP-ribosyltransferase	203			CHI14	YP_009609344.1	100	99.01

gp	locus tag	Description	Amino acids	Domains	pfam number	Closest homologue			
						Phage	locus tag	q cov %	aa id %
20	UYM28673.1	hypothetical protein	56			CHI14	YP_009609345.1	100	96.36
21	UYM28674.1	hypothetical protein	42			Virsaitis27	UJJ22005.1	100	100
22	UYM28675.1	hypothetical protein	101			CHI14	YP_009609348.1	69	100
23	UYM28676.1	small outer capsid protein	84	Small outer capsid protein	PF16855	Virsaitis27	UJJ22008.1	100	98.8
24	UYM28677.1	dCTPase	173			Q7622	UIS65546.1	100	90.29
25	UYM28678.1	hypothetical protein	112			X20	YP_010092176.1	100	96.4
26	UYM28679.1	MazG domain-containing protein	95			X20	YP_010092177.1	100	100
27	UYM28680.1	hypothetical protein	39			CHI14	YP_009609353.1	100	87.18
28	UYM28681.1	DNA primase	341			X20	YP_010092179.1	100	99.71
29	UYM28682.1	hypothetical protein	156	Major capsid protein Gp23	PF07068	CHI14	YP_009609355.1	100	100
30	UYM28683.1	spackle periplasmic protein	105			Kc304	QYN80476.1	100	99.04
31	UYM28684.1	DNA primase-helicase subunit	477	DnaB-like helicase C terminal domain	PF03796	CHI14	YP_009609357.1	100	100
32	UYM28685.1	capsid vertex assembly chaperone	115	Head assembly gene product	PF11113	Kc304	QYN80478.1	100	100
33	UYM28686.1	RecA-like recombination protein	389	RecA bacterial DNA recombination protein	PF00154	CHI14	YP_009609359.1	100	100
34	UYM28687.1	hypothetical protein	74			Kc304	QYN80480.1	100	100
35	UYM28688.1	hypothetical protein	183			CHI14	YP_009609361.1	100	100
36	UYM28689.1	putative RNA polymerase binding protein	304			CHI14	YP_009609362.1	100	99.67
37	UYM28690.1	putative thymidylate synthase	233	Thymidylate synthase	PF00303	Kc304	QYN80483.1	100	100
38	UYM28691.1	chorismate mutase domain-containing protein	115	Chorismate mutase type II	PF01817	CHI14	YP_009609364.1	100	100
39	UYM28692.1	putative peptidase	390	Peptidase family U32	PF01136	CHI14	YP_009609365.1	100	100
40	UYM28693.1	putative endonuclease	213	NUMOD3 motif	PF07460	Virsaitis27	UJJ22023.1	100	99.53
41	UYM28694.1	capsule biosynthesis protein	122			CHI14	YP_009609367.1	100	95.87

gp	locus tag	Description	Amino acids	Domains	pfam number	Closest homologue			
						Phage	locus tag	q cov %	aa id %
42	UYM28695.1	NTP transferase domain-containing protein	565	Nucleotidyl transferase	PF00483	Virsaitis27	UJJ22025.1	99	99.28
43	UYM28696.1	hypothetical protein	48			Kc304	QYN80488.1	100	97.87
44	UYM28697.1	D-arabinose-5-phosphate isomerase	209	SIS domain	PF01380	Kc304	QYN80489.1	100	99.52
45	UYM28698.1	hypothetical protein	140			Virsaitis27	UJJ22028.1	100	99.28
46	UYM28699.1	DNA polymerase	903	DNA polymerase family B, exonuclease domain; DNA polymerase family B	PF03104; PF00136	Virsaitis27	UJJ22029.1	100	99.78
47	UYM28700.1	translational repressor RegA	121	Bacteriophage translational regulator	PF01818	CHI14	YP_009609373.1	100	100
48	UYM28701.1	DNA polymerase clamp loader subunit	192	Bacteriophage clamp loader A subunit	PF16790	Kc304	QYN80493.1	100	100
49	UYM28702.1	replication factor C small subunit DNA polymerase clamp loader subunit	324	ATPase family associated with various cellular activities (AAA)	PF00004	X20	YP_010092200.1	100	99.69
50	UYM28703.1	sliding clamp protein	230	DNA polymerase processivity factor; Gp45 sliding clamp, C terminal	PF02916; PF09116	CHI14	YP_009609376.1	100	99.56
51	UYM28704.1	RNA polymerase binding protein	124	Phage RNA polymerase binding, RpbA	PF10789	Kc304	QYN80496.1	100	98.37
52	UYM28705.1	hypothetical protein	64	Phage gene product 45.2	PF17470	CHI14	YP_009609378.1	100	100
53	UYM28706.1	recombination-related endonuclease	561	AAA domain	PF13476	Virsaitis27	UJJ22036.1	100	100
54	UYM28707.1	hypothetical protein	72	Family of unknown function (DUF5487)	PF17589	EcS1	YP_010090747.1	100	83.1
55	UYM28708.1	recombination-related endonuclease	340	Calcineurin-like phosphoesterase	PF00149	EcS1	YP_010090748.1	99	80.47
56	UYM28709.1	homing endonuclease	211			EI	QEM42259.1	87	68.31
57	UYM28710.1	hypothetical protein	88			X20	YP_010092206.1	100	98.85
58	UYM28711.1	hypothetical protein	117	Protein of unknown function (DUF2654)	PF10849	CHI14	YP_009609385.1	100	100

gp	locus tag	Description	Amino acids	Domains	pfam number	Closest homologue			
						Phage	locus tag	q cov %	aa id %
59	UYM28712.1	hypothetical protein	76	Family of unknown function (DUF5491)	PF17595	CHI14	YP_009609386.1	100	100
60	UYM28713.1	sigma factor for late transcription	179			X20	YP_010092210.1	100	100
61	UYM28714.1	hypothetical protein	88			X20	YP_010092211.1	100	98.85
62	UYM28715.1	hypothetical protein	77			X20	YP_010092212.1	100	100
63	UYM28716.1	hypothetical protein	114			X20	YP_010092213.1	100	100
64	UYM28717.1	hypothetical protein	87			X20	YP_010092214.1	100	97.67
65	UYM28718.1	glutaredoxin	104			CHI14	YP_009609392.1	100	100
66	UYM28719.1	hypothetical protein	91			X20	YP_010092216.1	100	100
67	UYM28720.1	hypothetical protein	81			CHI14	YP_009609394.1	100	100
68	UYM28721.1	anaerobic NTP reductase large subunit	43			CHI14	YP_009609396.1	57	95.83
69	UYM28722.1	recombination endonuclease VII	158	Recombination endonuclease VII; T4 recombination endonuclease VII, dimerisation	PF02945; PF09124	Virsaitis27	UJJ22057.1	100	98.73
70	UYM28723.1	hypothetical protein	101			Kc304	QYN80516.1	100	99
71	UYM28724.1	inhibitor of host Lon protease	151	PinA peptidase inhibitor	PF10465	CHI14	YP_009609399.1	100	100
72	UYM28725.1	hypothetical protein	101			CHI14	YP_009609400.1	100	100
73	UYM28726.1	hypothetical protein	65			CHI14	YP_009609401.1	100	100
74	UYM28727.1	hypothetical protein	51			CHI14	YP_009609402.1	100	100
75	UYM28728.1	hypothetical protein	56			CHI14	YP_009609404.1	100	100
76	UYM28729.1	hypothetical protein	55			CHI14	YP_009609405.1	100	100
77	UYM28730.1	hypothetical protein	101			Kc304	QYN80525.1	100	100
78	UYM28731.1	hypothetical protein	110			CHI14	YP_009609408.1	100	99.08
79	UYM28732.1	hypothetical protein	172			Virsaitis27	UJJ22070.1	100	99.42
80	UYM28733.1	hypothetical protein	75			CHI14	YP_009609410.1	100	100

gp	locus tag	Description	Amino acids	Domains	pfam number	Closest homologue			
						Phage	locus tag	q cov %	aa id %
81	UYM28734.1	putative thioredoxin	79			CHI14	YP_009609411.1	100	98.72
82	UYM28735.1	hypothetical protein	347			Virsaitis27	UJJ22073.1	100	99.13
83	UYM28736.1	hypothetical protein	258			Virsaitis27	UJJ22074.1	100	97.67
84	UYM28737.1	hypothetical protein	69			Virsaitis27	UJJ22075.1	100	97.06
85	UYM28738.1	hypothetical protein	63			Kc304	QYN80533.1	100	98.39
86	UYM28739.1	hypothetical protein	123			Kc304	QYN80534.1	100	100
87	UYM28740.1	hypothetical protein	101			Kc304	QYN80535.1	99	94.95
88	UYM28741.1	hypothetical protein	156			Kc304	QYN80536.1	100	99.35
89	UYM28742.1	hypothetical protein	63			Kc304	QYN80537.1	100	98.39
90	UYM28743.1	hypothetical protein	162			Kc304	QYN80538.1	100	97.52
91	UYM28744.1	hypothetical protein	77			X20	YP_010092243.1	100	97.37
92	UYM28745.1	thioredoxin / AAA domain-containing protein	327			Kc304	QYN80540.1	100	100
93	UYM28746.1	hypothetical protein	74			X20	YP_010092246.1	100	100
94	UYM28747.1	thioredoxin	339	RNA repair pathway DNA polymerase beta family	PF10127	X20	YP_010092247.1	100	99.41
95	UYM28748.1	hypothetical protein	141			Virsaitis27	UJJ22086.1	100	100
96	UYM28749.1	hypothetical protein	80			Kc304	QYN80544.1	100	96.2
97	UYM28750.1	hypothetical protein	173			Virsaitis27	UJJ22088.1	100	95.93
98	UYM28751.1	hypothetical protein	162			Kc304	QYN80546.1	100	98.76
99	UYM28752.1	hypothetical protein	88			Virsaitis27	UJJ22090.1	100	98.85
100	UYM28753.1	hypothetical protein	76			CHI14	YP_009609429.1	100	100
101	UYM28754.1	hypothetical protein	229			Virsaitis27	UJJ22092.1	100	99.12
102	UYM28755.1	putative C4-type zinc finger containing protein	89	Prokaryotic dksA/traR C4-type zinc finger	PF01258	Kc304	QYN80550.1	100	100
103	UYM28756.1	hypothetical protein	44			Kc304	QYN80551.1	100	100

gp	locus tag	Description	Amino acids	Domains	pfam number	Closest homologue			
						Phage	locus tag	q cov %	aa id %
104	UYM28757.1	hypothetical protein	149			Kc304	QYN80552.1	100	98.65
105	UYM28758.1	hypothetical protein	129	Family of unknown function (DUF5856)	PF19174	X20	YP_010092258.1	100	99.22
106	UYM28759.1	lysis inhibition regulator membrane protein	98			CHI14	YP_009609435.1	100	98.97
107	UYM28760.1	hypothetical protein	71			CHI14	YP_009609436.1	100	100
108	UYM28761.1	hypothetical protein	128			CHI14	YP_009609437.1	100	99.21
109	UYM28762.1	hypothetical protein	57			CHI14	YP_009609438.1	100	100
110	UYM28763.1	Thymidine kinase	196	Thymidine kinase	PF00265	X20	YP_010092263.1	100	100
111	UYM28764.1	hypothetical protein	70			Virsaitis27	UJJ22102.1	100	98.55
112	UYM28765.1	hypothetical protein	59			Kc304	QYN80559.1	100	100
113	UYM28766.1	macro domain-containing protein	156	Macro domain	PF01661	Kc304	QYN80560.1	100	100
114	UYM28767.1	valyl-tRNA synthetase modifier	120			Kc304	QYN80561.1	100	99.16
115	UYM28768.1	hypothetical protein	183			CHI14	YP_009609443.1	100	100
116	UYM28769.1	site-specific RNA endonuclease	152	T4-page Endoribonuclease RegB	PF10715	CHI14	YP_009609444.1	100	98.68
117	UYM28770.1	hypothetical protein	92			CHI14	YP_009609446.1	100	98.75
118	UYM28771.1	hypothetical protein	81			CHI14	YP_009609446.1	100	98.75
119	UYM28772.1	hypothetical protein	89			CHI14	YP_009609447.1	100	100
120	UYM28773.1	terminase large subunit	178			Virsaitis27	UJJ22112.1	100	100
121	UYM28774.1	putative IPIII internal head protein	190			Kc304	QYN80569.1	100	98.41
122	UYM28775.1	endonuclease V	139	Pyrimidine dimer DNA glycosylase	PF03013	Kc304	QYN80570.1	100	97.83
123	UYM28776.1	hypothetical protein	96			CHI14	YP_009609450.1	100	100
124	UYM28777.1	lysozyme R	162	Phage lysozyme	PF00959	Kc304	QYN80572.1	100	99.38
125	UYM28778.1	nudix hydrolase	158	NUDIX domain	PF00293	CHI14	YP_009609452.1	100	98.09
126	UYM28779.1	hypothetical protein	79			CHI14	YP_009609453.1	100	100

gp	locus tag	Description	Amino acids	Domains	pfam number	Closest homologue			
						Phage	locus tag	q cov %	aa id %
127	UYM28780.1	hypothetical protein	110			Virsaitis27	UJJ22119.1	100	100
128	UYM28781.1	hypothetical protein	180			X20	YP_010092279.1	100	99.44
129	UYM28782.1	hypothetical protein	311			Kc304	QYN80577.1	100	100
130	UYM28783.1	hypothetical protein	91			Kc304	QYN80578.1	100	100
131	UYM28784.1	hypothetical protein	98			Kc304	QYN80579.1	100	98.97
132	UYM28785.1	hypothetical protein	124			Kc304	QYN80580.1	100	99.19
133	UYM28786.1	hypothetical protein	274			Kc304	QYN80581.1	100	98.9
134	UYM28787.1	hypothetical protein	202			Kc304	QYN80582.1	100	100
135	UYM28788.1	hypothetical protein	132			Kc304	QYN80583.1	100	99.24
136	UYM28789.1	hypothetical protein	188			Virsaitis27	UJJ22129.1	100	99.47
137	UYM28790.1	hypothetical protein	121			Kc304	QYN80585.1	100	94.17
138	UYM28791.1	hypothetical protein	150	Bacteriophage protein GP30.3	PF08010	CHI14	YP_009609465.1	100	96.64
139	UYM28792.1	hypothetical protein	75			X20	YP_010092291.1	100	98.65
140	UYM28793.1	hypothetical protein	79			CHI14	YP_009609467.1	100	100
141	UYM28794.1	hypothetical protein	76			Virsaitis27	UJJ22134.1	100	97.33
142	UYM28795.1	hypothetical protein	82			Virsaitis27	UJJ22135.1	100	100
143	UYM28796.1	Yad_anchor domain-containing protein	126	YadA-like membrane anchor domain	PF03895	CHI14	YP_009609470.1	92	96.52
144	UYM28797.1	hypothetical protein	62			Kc304	QYN80590.1	100	98.36
145	UYM28798.1	hypothetical protein	62			X20	YP_010092297.1	100	100
146	UYM28799.1	hypothetical protein	111			CHI14	YP_009609474.1	100	100
147	UYM28800.1	hypothetical protein	152			CHI14	YP_009609475.1	100	99.34
148	UYM28801.1	chaperone for long tail fiber formation	90			X20	YP_010092300.1	100	98.88
149	UYM28802.1	dNMP kinase	231			Virsaitis27	UJJ22142.1	100	99.57

gp	locus tag	Description	Amino acids	Domains	pfam number	Closest homologue			
						Phage	locus tag	q cov %	aa id %
150	UYM28803.1	tail completion and sheath stabiliser protein	196	T4-like virus tail tube protein Gp19	PF06841	CHI14	YP_009609477.1	100	100
151	UYM28804.1	putative mobile endonuclease	219			Kc304	QYN80597.1	100	99.54
152	UYM28805.1	DNA end protector protein	277			X20	YP_010092304.1	100	100
153	UYM28806.1	head completion protein	150	TnsA-like endonuclease N terminal	PF08722	CHI14	YP_009609479.1	100	100
154	UYM28807.1	baseplate wedge subunit	192	Base plate wedge protein 53	PF11246	CHI14	YP_009609480.1	100	100
155	UYM28808.1	baseplate hub subunit and tail lysozyme	576	Gp5 N-terminal OB domain; Phage lysozyme; Gp5 C-terminal repeat (multiple copies)	PF06714; PF00959; PF06715	Virsaitis27	UJJ22147.1	100	100
156	UYM28809.1	hypothetical protein	169			Kc304	QYN80602.1	100	100
157	UYM28810.1	hypothetical protein	99			X20	YP_010092309.1	100	97.96
158	UYM28811.1	putative endonuclease	225	GIY-YIG catalytic domain; NUMOD3 motif	PF01541; PF07460	X20	YP_010092310.1	100	98.66
159	UYM28812.1	baseplate wedge subunit	651			CHI14	YP_009609483.1	100	100
160	UYM28813.1	baseplate wedge initiator	1029			Kc304	QYN80605.1	100	99.32
161	UYM28814.1	baseplate wedge subunit	335	Bacteriophage T4, Gp8	PF09215	Kc304	QYN80606.1	100	100
162	UYM28815.1	baseplate wedge tail fiber connector	291	Bacteriophage T4 Gp9/10-like protein	PF07880	CHI14	YP_009609486.1	100	100
163	UYM28816.1	baseplate wedge subunit and tail pin	610	Bacteriophage T4 Gp9/10-like protein	PF07880	X20	YP_010092315.1	100	99.84
164	UYM28817.1	baseplate wedge subunit and tail pin	228	Gp11 baseplate wedge protein	PF08677	Virsaitis27	UJJ22155.1	100	99.56
165	UYM28818.1	short tail fiber protein	515	Phage short tail fibre protein Gp12, middle domain; Phage Tail Collar Domain; Short tail fibre protein receptor-binding domain	PF09089; PF07484; PF14928	X20	YP_010092317.1	100	100
166	UYM28819.1	fibritin neck whisker protein	585	Fibritin C-terminal region	PF07921	CHI14	YP_009609490.1	100	100

gp	locus tag	Description	Amino acids	Domains	pfam number	Closest homologue			
						Phage	locus tag	q cov %	aa id %
167	UYM28820.1	neck protein	313			Kc304	QYN80612.1	100	99.68
168	UYM28821.1	neck protein	259	Virus neck protein	PF11649	Virsaitis27	UJJ22159.1	100	99.22
169	UYM28822.1	Mob-like putative homing endonuclease	199			Merci	UGO49532.1	100	80.81
170	UYM28823.1	tail sheath stabiliser and completion protein	262	T4-like virus Myoviridae tail sheath stabiliser	PF16724	X20	YP_010092321.1	100	100
171	UYM28824.1	terminase DNA packaging enzyme small subunit	167	Terminase DNA packaging enzyme	PF11053	CHI14	YP_009609494.1	100	99.4
172	UYM28825.1	terminase large subunit	612	Terminase large subunit, T4likevirus-type, N-terminal; Terminase RNaseH-like domain	PF03237; PF17289	CHI14	YP_009609495.1	100	100
173	UYM28826.1	tail sheath protein	661	Phage tail sheath protein beta-sandwich domain; Phage tail sheath protein subtilisin-like domain; Phage tail sheath C-terminal domain	PF17481; PF04984; PF17482	CHI14	YP_009609496.1	100	99.85
174	UYM28827.1	hypothetical protein	228			CHI14	YP_009609497.1	100	100
175	UYM28828.1	tail tube protein	164	T4-like virus tail tube protein Gp19	PF06841	CHI14	YP_009609498.1	100	100
176	UYM28829.1	portal protein	523	Bacteriophage T4-like portal protein (Gp20)	PF07230	Virsaitis27	UJJ22165.1	100	100
177	UYM28830.1	prohead core protein	88	Gene product 67	PF17634	Kc304	QYN80620.1	51	100
178	UYM28831.1	capsid and scaffold protein	142			Kc304	QYN80621.1	100	100
179	UYM28832.1	prohead core scaffolding protein and protease	216	Prohead core protein serine protease	PF03420	CHI14	YP_009609502.1	100	100
180	UYM28833.1	prohead core scaffold protein	272			CHI14	YP_009609503.1	100	99.63
181	UYM28834.1	major capsid protein	520	Major capsid protein Gp23	PF07068	CHI14	YP_009609504.1	100	100
182	UYM28835.1	capsid vertex protein	418	Major capsid protein Gp23	PF07068	X20	YP_010092334.1	100	99.04
183	UYM28836.1	hypothetical protein	184			Kc304	QYN80626.1	100	98.91

gp	locus tag	Description	Amino acids	Domains	pfam number	Closest homologue			
						Phage	locus tag	q cov %	aa id %
184	UYM28837.1	RNA ligase 2	336	RNA ligase; T4 RNA ligase 2 C-terminal	PF09414; PF18043	CHI14	YP_009609507.1	100	97.91
185	UYM28838.1	hypothetical protein	76	Protein of unknown function (DUF2774)	PF11242	X20	YP_010092338.1	97	93.15
186	UYM28839.1	head outer capsid	180			X20	YP_010092339.1	100	98.88
187	UYM28840.1	inhibitor of prohead protease	237			X20	YP_010092340.1	100	99.15
188	UYM28841.1	RNA-DNA and DNA-DNA helicase	501	DEAD/DEAH box helicase; Helicase conserved C-terminal domain	PF00270; PF00271	X20	YP_010092341.1	100	99.6
189	UYM28842.1	RNA-DNA and DNA-DNA helicase	76	UvsW.1 domain	PF11637	CHI14	YP_009609513.1	100	100
190	UYM28843.1	DUF2685 domain-containing protein	55	Protein of unknown function (DUF2685)	PF10886	CHI14	YP_009609514.1	100	100
191	UYM28844.1	recombination repair and ssDNA binding protein	145	Recombination, repair and ssDNA binding protein UvsY	PF11056	CHI14	YP_009609515.1	100	100
192	UYM28845.1	baseplate wedge subunit	132	Baseplate wedge protein Gp25	PF04965	CHI14	YP_009609516.1	100	100
193	UYM28846.1	baseplate hub subunit	210	T4 bacteriophage base plate protein	PF12322	CHI14	YP_009609517.1	100	100
194	UYM28847.1	basepate hub assembly protein	251	T4 bacteriophage base plate protein	PF12322	Virsaitis27	UJJ22184.1	100	100
195	UYM28848.1	baseplate hub subunit	407	Baseplate structural protein, domain 1; Baseplate structural protein, domain 2	PF09097; PF09096	CHI14	YP_009609519.1	100	99.51
196	UYM28849.1	baseplate hub distal subunit	162	Baseplate hub distal subunit	PF11110	Virsaitis27	UJJ22186.1	100	98.14
197	UYM28850.1	baseplate hub tail length determinator	582			X20	YP_010092350.1	100	97.25
198	UYM28851.1	baseplate tail tube cap	367	Tail-tube assembly protein	PF11091	CHI14	YP_009609522.1	100	100
199	UYM28852.1	baseplate subunit	322			CHI14	YP_009609523.1	100	99.69
200	UYM28853.1	hypothetical protein	104	Family of unknown function (DUF5498)	PF17602	Virsaitis27	UJJ22190.1	100	100
201	UYM28854.1	RNA polymerase ADP-ribosylase	700	ADP-ribosyltransferase exoenzyme	PF03496	Virsaitis27	UJJ22191.1	100	97.85

gp	locus tag	Description	Amino acids	Domains	pfam number	Closest homologue			
						Phage	locus tag	q cov %	aa id %
202	UYM28855.1	hypothetical protein	61			CHI14	YP_009609526.1	100	98.33
203	UYM28856.1	DNA ligase	499	ATP dependent DNA ligase domain	PF01068	Virsaitis27	UJJ22193.1	100	99
204	UYM28857.1	hypothetical protein	89	Protein of unknown function (DUF3045)	PF11243	CHI14	YP_009609528.1	100	100
205	UYM28858.1	5'(3')-deoxyribonucleotidase	248			Kc304	QYN80649.1	100	100
206	UYM28859.1	hypothetical protein	69			X20	YP_010092361.1	100	100
207	UYM28860.1	hypothetical protein	56			CHI14	YP_009609532.1	100	98.18
208	UYM28861.1	hypothetical protein	149			CHI14	YP_009609533.1	100	100
209	UYM28862.1	hypothetical protein	119	Phage Gp30.7 protein	PF06919	CHI14	YP_009609534.1	100	98.31
210	UYM28863.1	hypothetical protein	175			X20	YP_010092365.1	100	95.98
211	UYM28864.1	hypothetical protein	157			CHI14	YP_009609536.1	100	98.08
212	UYM28865.1	hypothetical protein	123	Phage Gp30.8 protein	PF06019	Virsaitis27	UJJ22204.1	100	99.18
213	UYM28866.1	hypothetical protein	59			CHI14	YP_009609538.1	100	100
214	UYM28867.1	rIII lysis inhibitor accessory protein	83			CHI14	YP_009609539.1	100	100
215	UYM28868.1	head assembly cochaperone	108	Chaperonin 10 Kd subunit	PF00166	CHI14	YP_009609540.1	100	100
216	UYM28869.1	putative tail fibers protein	108	WYL_2, Sm-like SH3 beta-barrel fold	PF10902	CHI14	YP_009609541.1	100	100
217	UYM28870.1	dCMP deaminase	188	MafB19-like deaminase	PF14437	Kc304	QYN80661.1	100	100
218	UYM28871.1	hypothetical protein	114			CHI14	YP_009609543.1	100	100
219	UYM28872.1	2-keto-3-deoxy-D-arabino-heptulosonate-7-phosphate synthase I alpha	339	DAHP synthetase I family	PF00793	Kc304	QYN80663.1	100	98.52
220	UYM28873.1	hypothetical protein	77			X20	YP_010092375.1	100	98.68
221	UYM28874.1	hypothetical protein	89			Kc304	QYN80665.1	100	98.86
222	UYM28875.1	polynucleotide kinase/phosphatase	300	AAA domain	PF13671	Kc304	QYN80666.1	100	99.67
223	UYM28876.1	hypothetical protein	73			Kc304	QYN80667.1	100	100

gp	locus tag	Description	Amino acids	Domains	pfam number	Closest homologue			
						Phage	locus tag	q cov %	aa id %
224	UYM28877.1	hypothetical protein	162			Kc304	QYN80668.1	100	98.76
225	UYM28878.1	outer membrane lipoprotein Rz1	100			Kc304	QYN80669.1	100	100
226	UYM28879.1	putative membrane protein	119			Kc304	QYN80670.1	100	100
227	UYM28880.1	inhibitor of host transcription	167	Phage ALC protein	PF17527	X20	YP_010092382.1	100	100
228	UYM28881.1	RNA ligase 1	391	RNA ligase	PF09511	Kc304	QYN80672.1	100	100
229	UYM28882.1	endonuclease II	138	GIY-YIG catalytic domain	PF01541	CHI14	YP_009609554.1	100	99.27
230	UYM28883.1	ribonucleoside-diphosphate reductase subunit beta	393	Ribonucleotide reductase, small chain	PF00268	Kc304	QYN80674.1	100	98.98
231	UYM28884.1	hypothetical protein	66			CHI14	YP_009609556.1	100	100
232	UYM28885.1	ribonucleotide reductase alpha subunit	752	ATP cone domain; Ribonucleotide reductase, all-alpha domain; Ribonucleotide reductase, barrel domain	PF03477; PF00317; PF02867	CHI14	YP_009609557.1	100	99.87
233	UYM28886.1	thymidylate synthase	287	Thymidylate synthase	PF00303	Virsaitis27	UJJ22229.1	100	99.65
234	UYM28887.1	hypothetical protein	125			CHI14	YP_009609560.1	100	100
235	UYM28888.1	hypothetical protein	103			X20	YP_010092390.1	96	100
236	UYM28889.1	dihydrofolate reductase	201	Dihydrofolate reductase	PF00186	CHI14	YP_009609562.1	100	98
237	UYM28890.1	hypothetical protein	75			CHI14	YP_009609563.1	100	98.65
238	UYM28891.1	hypothetical protein	93	Family of unknown function (DUF5417)	PF17438	X20	YP_010092393.1	100	98.91
239	UYM28892.1	hypothetical protein	78			CHI14	YP_009609566.1	100	100
240	UYM28893.1	hypothetical protein	92			CHI14	YP_009609567.1	81	98.65
241	UYM28894.1	hypothetical protein	71			CHI14	YP_009609568.1	100	98.57
242	UYM28895.1	single stranded DNA binding protein	296	Gp32 DNA binding protein like	PF08804	CHI14	YP_009609569.1	95	100

gp	locus tag	Description	Amino acids	Domains	pfam number	Closest homologue			
						Phage	locus tag	q cov %	aa id %
243	UYM28896.1	loader of DNA helicase	218	T4 gene Gp59 loader of gp41 DNA helicase; T4 gene Gp59 loader of gp41 DNA helicase C-term	PF08993; PF08994	CHI14	YP_009609570.1	100	100
244	UYM28897.1	homing endonuclease	198			CHI14	YP_009609571.1	100	97.52
245	UYM28898.1	late promoter transcription accessory protein	104	Phage late-transcription coactivator	PF16805	CHI14	YP_009609572.1	100	100
246	UYM28899.1	dsDNA binding protein	96	Transcriptional regulator DsbA	PF11126	CHI14	YP_009609573.1	86	1000
247	UYM28900.1	ribonuclease	314	5'-3' exonuclease, N-terminal resolvase-like domain; T4 RNase H, C terminal	PF02739; PF09293	CHI14	YP_009609574.1	100	100
248	UYM28901.1	long tail fiber proximal subunit	1257			CHI14	YP_009609575.1	100	99.76
249	UYM28902.1	hinge connector of long tail fiber proximal connector	384			X20	YP_010092404.1	100	99.74
250	UYM28903.1	hinge connector of long tail fiber proximal connector	220			CHI14	YP_009609577.1	100	99.54
251	UYM28904.1	long tail fiber distal subunit	915	Chaperone of endosialidase	PF13884	CBH8	ARW57951.1	100	98.03
252	UYM28905.1	tail fiber protein	172	Phage tail fibre adhesin Gp38	PF05268	Kc304	QYN80697.1	100	100
253	UYM28906.1	tail fiber protein	169	Phage tail fibre adhesin Gp38	PF05268	Kc304	QYN80698.1	100	100
254	UYM28907.1	holin	219	Bacteriophage T holin	PF11031	CHI14	YP_009609581.1	100	100
255	UYM28908.1	anti-sigma 70 protein	90	Anti-Sigma Factor A	PF09010	Virsaitis27	UJJ22252.1	100	98.88
256	UYM28909.1	hypothetical protein	113			Kc304	QYN80701.1	100	98.21
257	UYM28910.1	hypothetical protein	73			Virsaitis27	UJJ22255.1	100	100
258	UYM28911.1	hypothetical protein	99			Kc304	QYN80703.1	100	98.98
259	UYM28912.1	hypothetical protein	145			Kc304	QYN80704.1	100	99.31
260	UYM28913.1	hypothetical protein	116			Kc304	QYN80705.1	100	99.13

gp	locus tag	Description	Amino acids	Domains	pfam number	Closest homologue			
						Phage	locus tag	q cov %	aa id %
261	UYM28914.1	MotA activator of middle period transcription	212	Transcription factor MotA, activation domain; Bacteriophage T4 MotA, C-terminal	PF09114; PF09158	CHI14	QYN80706.1	100	100
262	UYM28915.1	hypothetical protein	49			Kc304	QYN80707.1	100	97.92
263	UYM28916.1	hypothetical protein	147			X20	YP_010092417.1	90	100
264	UYM28917.1	hypothetical protein	47			CHI14	YP_009609591.1	58	100
265	UYM28918.1	DNA topoisomerase	455	DNA gyrase/topoisomerase IV, subunit A	PF00521	Kc304	QYN80709.1	100	99.78
266	UYM28919.1	hypothetical protein	52			Kc304	QYN80710.1	100	100
267	UYM28920.1	hypothetical protein	58			X20	YP_010092421.1	100	100
268	UYM28921.1	nucleoid disruption protein	149	T4-like phage nuclear disruption protein (Ndd)	PF06591	Kc304	QYN80712.1	100	98.65
269	UYM28922.1	hypothetical protein	67			CHI14	YP_009609595.1	100	98.48
270	UYM28923.1	hypothetical protein	41			Kc304	QYN80714.1	55	100
271	UYM28924.1	endonuclease IV	197			Kc304	QYN80715.1	100	100
272	UYM28925.1	hypothetical protein	101			CHI14	YP_009609598.1	100	100
273	UYM28926.1	hypothetical protein	67			CHI14	YP_009609599.1	100	100
274	UYM28927.1	protector from prophage-induced early lysis (rIIB)	310			CHI14	YP_009609600.1	100	99.68

Full names of phages encoding the closest homologues: *Serratia* phage CHI14, *Serratia* phage X20, *Serratia* phage CBH8, *Kosakonia* phage Kc304, *Erwinia* phage Virsaitis27, *Klebsiella* phage Ei, *Klebsiella* phage Merci, *Escherichia* phage EcS1 and *Enterobacter* phage Q7622

Table S5: T4-like core genes and their homologues in LC53.

T4-like core genes		LC53 homologues		Identity	
**Name**	**Description/Function**	**gp**	**Locus tag**	**q cov %**	**aa id %**
DNA replication, repair and recombination					
gp 43	DNA polymerase	46	UYM28699.1	99	64
gp 45	trimeric sliding clamp	50	UYM28703.1	100	64
gp 44	sliding clamp loader complex	49	UYM28702.1	99	70
gp 62	sliding clamp loader complex	48	UYM28701.1	100	55.5
gp 41	helicase-primase complex	31	UYM28684.1	81	70
gp 61	helicase-primase complex	28	UYM28681.1	100	59.5
gp 59	helicase-primase loader	1	UYM28896.1	99	74
gp 32	single-strand binding protein	242	UYM28895.1	100	64.5
gp 46	subunit of recombination nuclease ceomplex required for initiation of DNA replication	53	UYM28706.1	99	75
gp 47	subunit of recombination nuclease ceomplex required for initiation of DNA replication	55	UYM28708.1	99	70
uvsW	recombination DNA-RNA helicase, DNA dependent ATPase	188	UYM28841.1	85	75
uvsX	RecA-like recombination protein	33	UYM28686.1	98	79.5
uvsY	usvX helper protein	191	UYM28844.1	99	71
gp 30	DNA ligase	203	UYM28856.1	100	56.5
rnh	Ribonuclease H	247	UYM28900.1	100	55
gp 39	subunit of a Type II DNA topoisomerase	5	UYM28658.1	91	77.5
gp 60	subunit of a Type II DNA topoisomerase	5	UYM28658.1	14	64
gp 52	subunit of a Type II DNA topoisomerase	265	UYM28918.1	99	75
dda	short-range DNA helicase	16	UYM28669.1	100	62
gp 49	Endonuclease VII, required for recombination and DNA packaging	69	UYM28722.1	100	77.5

T4-like core genes		LC53 homologues		Identity	
**Name**	**Description/Function**	**gp**	**Locus tag**	**q cov %**	**aa id %**
Auxiliary metabolism					
nrdA	subunit of an aerobic ribonucleotide reductase complex	232	UYM28885.1	99	77.5
nrdB	subunit of an aerobic ribonucleotide reductase complex	230	UYM28883.1	37	74
nrdC	subunit of an anaerobic ribonucleotide reductase complex	65	UYM28718.1	80	23.5
nrdG	subunit of an anaerobic ribonucleotide reductase complex	absent			
nrdH	thioredoxin	65	UYM28718.1	100	63
gp 56	dCTPase-dUTPase	24	UYM28677.1	100	64
cd	dCMP deaminase	217	UYM28870.1	99	69.5
frd	dihydrofolate	236	UYM28889.1	99	51.5
td	thymidylate synthase	233	UYM28886.1	45	81
tk	thymidine kinase	110	UYM28763.1	97	67.5
gp 1	dNMP kinase	149	UYM28802.1	98	50.5
denA	Endonuclease II	229	UYM28882.1	94	75
dexA	Exonuclease A	14	UYM28667.1	100	65
Gene expression					
gp 33	essential protein that mediates Gp55-Gp45-RNA polymerase interactions in late transcription	245	UYM28898.1	100	60
gp 55	sigma factor for late transcription	60	UYM28713.1	94	76
regA	mRNA-binding translational repressor	47	UYM28700.1	99	76
Phage morphogenesis					
gp 2	protection of packaged DNA against RecBCD nuclease	152	UYM28805.1	99	67.5
gp 3	sheath terminator	150	UYM28803.1	94	53
gp 4	head completion protein	153	UYM28806.1	99	68

T4-like core genes		LC53 homologues		Identity	
Name	Description/Function	gp	Locus tag	q cov %	aa id %
Phage morphogenesis					
gp 5	baseplate lysozyme hub component	155	UYM28808.1	99	69
gp 6	baseplate wedge component	159	UYM28812.1	100	69.5
gp 8	baseplate wedge	161	UYM28814.1	100	73
gp 13	head completion protein	167	UYM28820.1	100	74.5
gp 14	head completion protein	168	UYM28821.1	100	71
gp 15	tail completion protein	170	UYM28823.1	94	60.5
gp 16	subunit of terminase for DNA packaging	171	UYM28824.1	97	75.5
gp 17	subunit of terminase for DNA packaging	172	UYM28825.1	100	81.5
gp 18	tail sheath subunit	173	UYM28826.1	99	72
gp 19	tail tube subunit	175	UYM28828.1	100	72
gp 20	head portal vertex protein	176	UYM28829.1	100	75
gp 21	prohead core protein and protease	179	UYM28832.1	98	83
gp 22	prohead core protein	180	UYM28833.1	100	63
gp 23	precursor of major capsid protein	181	UYM28834.1	99	85.5
gp 25	baseplate wedge subunit	192	UYM28845.1	97	72
gp 26	baseplate hub subunit	193	UYM28846.1	99	55
gp 34	proximal tail fibre protein subunit	248	UYM28901.1	100	49
gp 35	tail fibre hinge protein	249	UYM28902.1	99	34
gp 36	small distal tail fibre protein subunit	250	UYM28903.1	100	38
gp 37	large distal tail fibre protein subunit	251	UYM28904.1	17	32.5
gp 49	Endonuclease VII, required for DNA packaging	69	UYM28722.1	100	77.5
gp 53	baseplate wedge component	154	UYM28807.1	97	61

T4-like core genes		LC53 homologues		Identity	
**Name**	**Description/Function**	**gp**	**Locus tag**	**q cov %**	**aa id %**
Others					
rIIA		1	UYM28654.1	99	35.5
rIIB		274	UYM28927.1	99	54

Bold genes are T4-like core genes and unbolded genes are quasi core genes.

Table S6: Comparison of transcriptional organisation to phage T4.

Protein in T4	Homologue in LC53	q cov %	aa id %	Description and function
Alc	Gp227	99	46	Modification of host RNA polymerase residues to further increase early phage transcription via inhibition of host transcription
AsiA	Gp255	92	57	Sigma appropriation – conversion of early to middle transcription via activation of MotA containing middle promoters
MotA	Gp261	95	50	Sigma appropriation – conversion of early to middle transcription via activation of MotA containing middle promoters
ComC-α	Gp10 and Gp11	Nd	Nd	Downstream extension of early transcripts by increasing stability of synthesized RNA
ModB	Gp19	84	43	ADP ribosylation enzyme involved in conversion from early to middle transcription
DenA	Gp246	89	64	Endonuclease II degrading host DNA during early and middle transcription to remove competing templates for host RNA polymerase
DenB	Gp271	87	53	Endonuclease IV degrading host DNA during early and middle transcription to remove competing templates for host RNA polymerase
Gp45	Gp50	100	64	Activator of late transcription
Gp55	Gp60	94	76	Activator of late transcription
Gp33	Gp245	100	60	Co-activator of late transcription

Table S7: LC53 genomic loci downstream of the phage T4 conserved promoter sequence 5’-TATAAATA-3’.

CDS in proximity	gp	Distance to start codon of the gene (in bp)	strand
soc	23	-31	template
tRNA-Lys		-10	template
tRNA-Tyr		-14	template
head completion protein	153	-10	coding
head completion protein	153	-27	coding
baseplate wedge tail fibre connector	162	-28	coding
tail sheath protein	173	-32	coding
portal protein	176	-41	coding
prohead core scaffold protein	180	-29	coding
capsid vertex protein	182	-27	coding
inhibitor of prohead protease	187	-33	coding
RNA-DNA and DNA-DNA helicase	188	-29	coding
baseplate hub subunit	193	-22	coding
baseplate hub assembly protein	194	-17	coding
long tail fibre proximal subunit	248	-33	coding
hinge connector	249	-24	coding
holin	254	-24	coding

Loci where the sequence is not upstream of a CDS are not mentioned.

Table S8: Comparison of DNA packaging mechanism to T4.

Protein in T4	Homologue in LC53	q cov %	aa id %	Description and function
small terminase	Gp171	97	76	DNA recognition, modulation of terminase complex
large terminase	Gp172	100	81	DNA packaging, ATPase activity, stimulated by presence of small terminase
Portal protein	Gp176	100	75	

Table S9: Nucleotide-based intergenomic similarities between Winklervirus phages and close relatives of other genera calculated with VIRIDIC (17) and protein identities of a defined set of core genes.

DNA / protein	LC53	Kc304	Virsaitis27	CHI14	CBH8	X20	EcS1	PEi20
**LC53**		94.7	93.83	93	93	927	66.4	49
**Kc304**	99.1		95.6	95.3	95.3	92.5	67.7	48.6
**Virsaitis27**	98.9	99.5		93.9	93.9	91.6	68.1	49
**CHI14**	98.9	99.7	99.3		99.99	94.7	68.3	49.2
**CBH8**	98.9	99.7	99.3	100		94.7	68.3	49.2
**X20**	98.9	98.9	98.7	99	99		67.7	48.5
**EcS1**	70.4	70.7	70.7	70.7	70.7	70.5		45.7
**PEi20**	65.3	65.4	65.3	65.4	65.4	65.1	64.1	

Table S10: CDS of LC53 for which no protein homologue was found in T4 and CDS of T4 for which no protein homologue was found in LC53.

Unique to LC53		Unique to T4	
**gp**	**Predicted function**	**protein name**	**function**
3	hypothetical protein	Vs.5 conserved protein	hypothetical protein
4	hypothetical protein	Vs.3 conserved protein	hypothetical protein
6	hypothetical protein	UvsY. 1 conserved protein	hypothetical protein
9	dCTP pyrophosphatase	Trna.3 conserved protein	hypothetical protein
10	comCalpha protein	Trna.2 conserved protein	hypothetical protein
11	comCalpha protein	Tk.2 conserved protein	hypothetical protein
13	transcription regulatory protein MotB	Tk.1 conserved protein	hypothetical protein
15	hypothetical protein	PseT.1 conserved protein	hypothetical protein
20	hypothetical protein	NrdC.9 conserved protein	hypothetical protein
21	hypothetical protein	NrdC.8 conserved protein	hypothetical protein
25	hypothetical protein	NrdC.7 conserved predicted membrane protein	hypothetical protein

Unique to LC53		Unique to T4	
**gp**	**Predicted function**	**protein name**	**function**
26	MazG domain containing protein	NrdC.6 conserved protein	hypothetical protein
27	hypothetical protein	NrdC.5 conserved protein	hypothetical protein
34	hypothetical protein	NrdC.4 conserved protein	hypothetical protein
35	hypothetical protein	NrdC.3 conserved protein	hypothetical protein
36	putative RNA polymerase binding protein	NrdA.2 conserved protein	hypothetical protein
38	chorismate mutase domain containing protein	Nrd.1 protein	hypothetical protein
39	putative peptidase	Ndd.6 conserved predicted outer membrane protein	hypothetical protein
41	capsule biosynthesis protein	Ndd.5 predicted outer membrane protein	hypothetical protein
42	NTP transferase domain containing protein	Ndd.4 predicted inner membrane	hypothetical protein
43	hypothetical protein	Ndd.3 predicted inner membrane protein	hypothetical protein
44	D-arabinose-5-phosphate isomerase	Ndd.2 predicted outer membrane protein	hypothetical protein
45	hypothetical protein	Mrh.2 protein	hypothetical protein
56	homing endonuclease	MotB.2 protein	hypothetical protein
57	hypothetical protein	MotB.1 protein	hypothetical protein
61	hypothetical protein	MotA.1 predicted periplasmic protein	hypothetical protein
64	hypothetical protein	ModA.4 protein	hypothetical protein
66	hypothetical protein	ModA.3 protein	hypothetical protein
67	hypothetical protein	MobD.5 protein	hypothetical protein
70	hypothetical protein	MobD.3 protein	hypothetical protein
73	hypothetical protein	MobD.2a protein	hypothetical protein
74	hypothetical protein	MobD.1 conserved protein	hypothetical protein
75	hypothetical protein	Gp61.4 protein	hypothetical protein
76	hypothetical protein	Gp61.2 protein	hypothetical protein
78	hypothetical protein	Gp60.1 protein	hypothetical protein
79	hypothetical protein	Gp55.8 conserved predicted membrane protein	hypothetical protein
80	hypothetical protein	Gp55.6 conserved protein	hypothetical protein
82	hypothetical protein	Gp55.4 conserved protein	hypothetical protein
83	hypothetical protein	Gp55.3 protein	hypothetical protein

Unique to LC53		Unique to T4	
**gp**	**Predicted function**	**protein name**	**function**
84	hypothetical protein	Gp52.1 conserved predicted membrane protein	hypothetical protein
85	hypothetical protein	Gp5.3 conserved protein	hypothetical protein
86	hypothetical protein	Gp49.3 protein	hypothetical protein
87	hypothetical protein	Gp47.1 predicted membrane protein	hypothetical protein
88	hypothetical protein	Gp46.2 protein	hypothetical protein
89	hypothetical protein	Gp31.2 protein	hypothetical protein
90	hypothetical protein	Gp30.6 conserved protein	hypothetical protein
91	hypothetical protein	Gp30.4 conserved protein	hypothetical protein
93	hypothetical protein	Gp30.3' protein	hypothetical protein
95	hypothetical protein	Gp24.3 conserved protein	hypothetical protein
96	hypothetical protein	Gp24.2 conserved protein	hypothetical protein
97	hypothetical protein	Frd.3 protein	hypothetical protein
98	hypothetical protein	Frd.2 conserved protein	hypothetical protein
99	hypothetical protein	e.8 conserved protein	hypothetical protein
100	hypothetical protein	e.7 protein	hypothetical protein
101	hypothetical protein	e.5 conserved protein	hypothetical protein
102	putative C4type zinc finger containing protein	e.2 conserved , predicted membrane protein	hypothetical protein
104	hypothetical protein	DexA.1 protein	hypothetical protein
106	lysis inhibition regulator membrane protein	Cd.5 protein	hypothetical protein
108	hypothetical protein	Cd.4 conserved protein	hypothetical protein
109	hypothetical protein	Cd.3 conserved protein	hypothetical protein
112	hypothetical protein	AsiA.1 protein	hypothetical protein
118	hypothetical protein	Alt. 3 conserved protein	hypothetical protein
123	hypothetical protein	a Gt.3 protein	hypothetical protein
126	hypothetical protein	a Gt.2 protein	hypothetical protein
127	hypothetical protein	superinfection immunity protein	protein superinfection immunity protein
128	hypothetical protein	sulfurtransferase	protein sulfurtransferase
129	hypothetical protein	sulfurtransferase	protein sulfurtransferase

Unique to LC53		Unique to T4	
**gp**	**Predicted function**	**protein name**	**function**
130	hypothetical protein	Stp activator of host PrrC lysyl tRNA endonuclease	protein Stp activator of host PrrC lysyl tRNA endonuclease
131	hypothetical protein	Srh transcription modulator	protein Srh transcription modulator
132	hypothetical protein	SegG homing endonuclease	protein SegG homing endonuclease
133	hypothetical protein	SegF homing endonuclease	protein SegF homing endonuclease
135	hypothetical protein	rI lysis inhibition regulator, membrane protein	protein rI lysis inhibition regulator, membrane protein
136	hypothetical protein	RepEB oriE replication initiation protein	protein RepEB oriE replication initiation protein
137	hypothetical protein	RepEA oriE replication initiation protein	protein RepEA oriE replication initiation protein
139	hypothetical protein	NrdC thioredoxin	protein NrdC thioredoxin
140	hypothetical protein	naphthalene 1,2 dioxygenase	protein naphthalene 1,2 dioxygenase
141	hypothetical protein	Mrh transcription modulator under heat shock	protein Mrh transcription modulator under heat shock
142	hypothetical protein	MotB modifier of transcription	protein MotB modifier of transcription
143	Yad anchor domain containing protein	molybdopterin guanine dinucleotide biosynthesis MobD	protein molybdopterin guanine dinucleotide biosynthesis protein MobD
144	hypothetical protein	ModA RNA polymerase ADP ribosylase	protein ModA RNA polymerase ADP ribosylase
146	hypothetical protein	IpII internal head protein	protein IpII internal head protein
148	chaperon for long tail fiber formation	IpI internal head protein	protein IpI internal head protein
169	Mob like putative homing endonuclease	iduronate sulfatase	protein iduronate sulfatase
174	hypothetical protein	I TevIII homing endonuclease (defective)	protein I TevIII homing endonuclease (defective)

Unique to LC53		Unique to T4	
**gp**	**Predicted function**	**protein name**	**function**
183	hypothetical protein	I TevII homing endonuclease	protein I TevII homing endonuclease
185	hypothetical protein	Gp57A chaperone for tail fiber formation	protein Gp57A chaperone for tail fiber formation
200	hypothetical protein	Gp55.5 conserved of unknown function	protein Gp55.5 conserved protein of unknown function
207	hypothetical protein	Gp49.1 conserved of unknown function	protein Gp49.1 conserved protein of unknown function
208	hypothetical protein	Gp38 distal long tail fiber assembly catalyst	protein Gp38 distal long tail fiber assembly catalyst
210	hypothetical protein	GoF mRNA metabolism modulator	protein goF mRNA metabolism modulator
211	hypothetical protein	GIY YIG nuclease family protein	protein GIY YIG nuclease family protein
219	2-keto-3-deoxy-D-arabinoheptulosonate-7-phosphate synthase I alpha	DUF4326 domain containing protein	protein DUF4326 domain containing protein
221	hypothetical protein	Dmd discriminator of mRNA degradation	protein Dmd discriminator of mRNA degradation
223	hypothetical protein	dextranase	protein dextranase
224	hypothetical protein	Dam family site specific DNA (adenine N6) methyltransferase	protein Dam family site specific DNA (adenine N6) methyltransferase
231	hypothetical protein	beta glucosyl transferase	protein b gt beta glucosyl transferase
234	hypothetical protein	autonomous glycyl radical cofactor GrcA	protein autonomous glycyl radical cofactor GrcA
235	hypothetical protein	Arn inhibitor of MrcBC restriction endonuclease (anti restriction nuclease)	protein Arn inhibitor of MrcBC restriction endonuclease (anti restriction nuclease)
237	hypothetical protein	anaerobic ribonucleoside triphosphate reductase activating protein	protein anaerobic ribonucleoside triphosphate reductase activating protein

Unique to LC53		Unique to T4	
**gp**	**Predicted function**	**protein name**	**function**
239	hypothetical protein	Ac acridine resistance protein	protein Ac acridine resistance protein
240	hypothetical protein	alpha glucosyl transferase	protein a gt alpha glucosyl transferase
241	hypothetical protein		
252	phage tail fibre adhesin		
253	phage tail fibre adhesin/ receptor recognising protein		
256	hypothetical protein		
262	hypothetical protein		
263	hypothetical protein		
264	hypothetical protein		
266	hypothetical protein		
267	hypothetical protein		
270	hypothetical protein		
272	hypothetical protein		

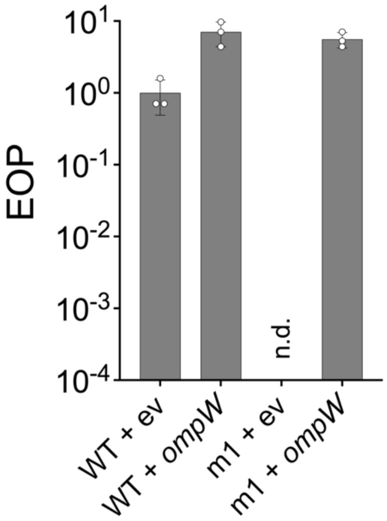

Figure S1: Complementation of *ompW* transposon mutant restores phage infectivity. EOP of *Serratia* sp. 39006 WT with empty vector (ev) or overexpressing *ompW* and *ompW* transposon mutant m1 with the empty vector or complemented with *ompW*. EOP for all the samples was calculated in comparison to the WT + ev control. Bars represent the average of three replicates and error bars show the standard deviation.

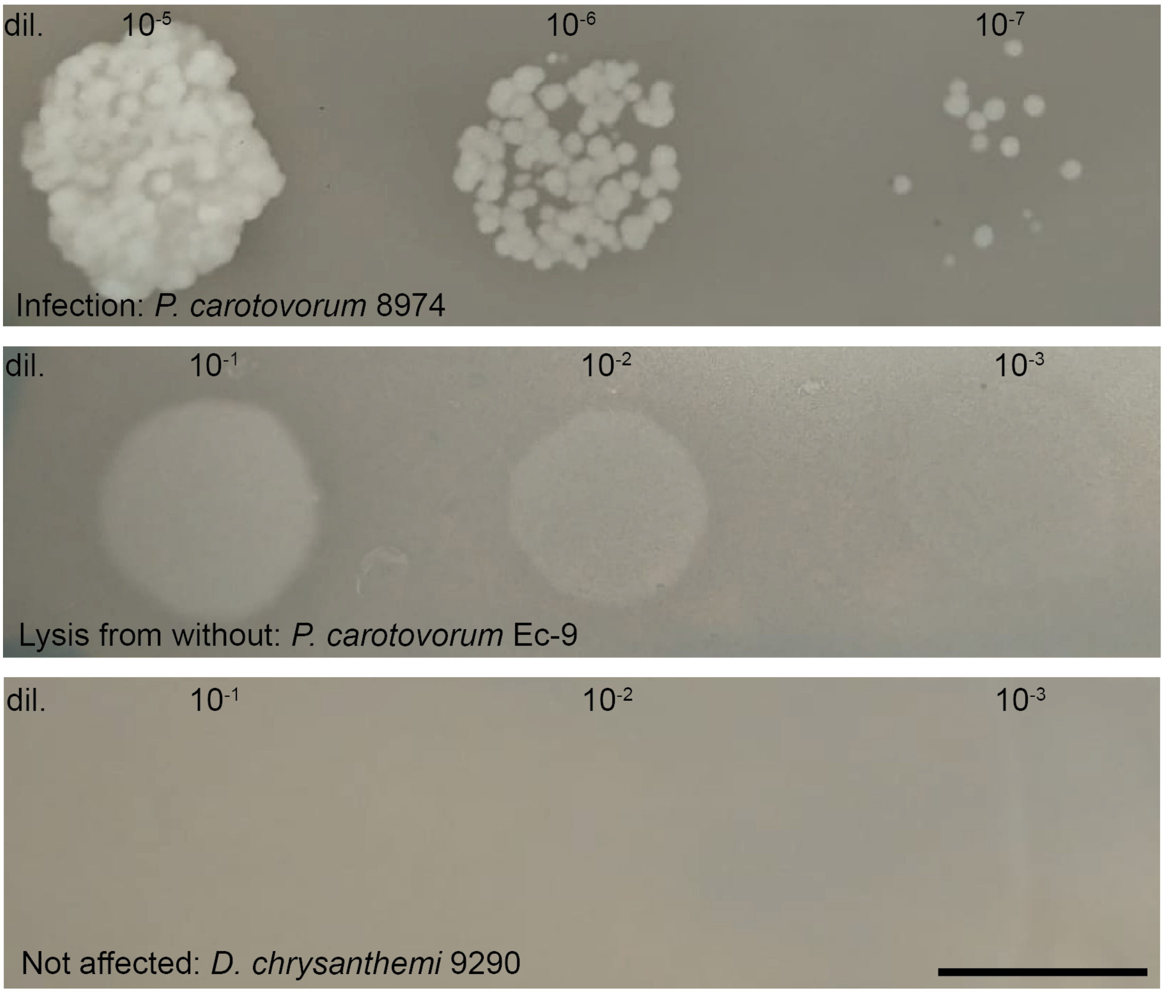

Figure S2: Examples for successful infection (top), visible as individual plaques, lysis from without (middle), visible as zones of less turbidity but no individual plaques and not affected (bottom) strains (scale bar: 1 cm).

References

1. Thoma S, Schobert M. An improved *Escherichia coli* donor strain for diparental mating. FEMS Microbiol Lett. 2009;294(2):127-32.
2. Jackson SA, Fellows BJ, Fineran PC. Complete Genome Sequences of the *Escherichia coli* Donor Strains ST18 and MFDpir. Microbiol Resour Announc. 2020;9(45).
3. Thomson NR, Crow MA, McGowan SJ, Cox A, Salmond GP. Biosynthesis of carbapenem antibiotic and prodigiosin pigment in *Serratia* is under quorum sensing control. Mol Microbiol. 2000;36(3):539-56.
4. Smith LM, Jackson SA, Malone LM, Ussher JE, Gardner PP, Fineran PC. The Rcs stress response inversely controls surface and CRISPR-Cas adaptive immunity to discriminate plasmids and phages. Nat Microbiol. 2021;6(2):162-72.
5. Palomar J, Guasch JF, Regue M, Vinas M. The effect of nuclease on transformation efficiency in *Serratia marcescens*. FEMS Microbiol Lett. 1990;57(3):255-8.
6. Rius N, Sole M, Francia A, Loren JG. Buffering Capacity of Pigmented and Nonpigmented Strains of *Serratia marcescens*. Appl Environ Microbiol. 1994;60(6):2152-4.
7. Bell KS, Avrova AO, Holeva MC, Cardle L, Morris W, De Jong W, et al. Sample sequencing of a selected region of the genome of *Erwinia carotovora* subsp. *atroseptica* reveals candidate phytopathogenicity genes and allows comparison with *Escherichia coli*. Microbiology (Reading). 2002;148(Pt 5):1367-78.
8. Fineran PC, Blower TR, Foulds IJ, Humphreys DP, Lilley KS, Salmond GP. The phage abortive infection system, ToxIN, functions as a protein-RNA toxin-antitoxin pair. Proc Natl Acad Sci U S A. 2009;106(3):894-9.
9. Bell KS, Sebaihia M, Pritchard L, Holden MT, Hyman LJ, Holeva MC, et al. Genome sequence of the enterobacterial phytopathogen *Erwinia carotovora* subsp. *atroseptica* and characterization of virulence factors. Proc Natl Acad Sci U S A. 2004;101(30):11105-10.
10. Tovkach FI. A Study of Erwinia carotovora Phage Resistance with the Use of Temperate Bacteriophage ZF40. Microbiology. 2002;71(1):72-8.
11. Birkholz N, Jackson SA, Fagerlund RD, Fineran PC. A mobile restriction-modification system provides phage defence and resolves an epigenetic conflict with an antagonistic endonuclease. Nucleic Acids Res. 2022;50(6):3348-61.
12. Portier P, Pedron J, Taghouti G, Fischer-Le Saux M, Caullireau E, Bertrand C, et al. Elevation of *Pectobacterium carotovorum* subsp. *odoriferum* to species level as *Pectobacterium odoriferum* sp. nov., proposal of *Pectobacterium brasiliense* sp. nov. and *Pectobacterium actinidiae* sp. nov., emended description of *Pectobacterium carotovorum* and description of *Pectobacterium versatile* sp. nov., isolated from streams and symptoms on diverse plants. Int J Syst Evol Microbiol. 2019;69(10):3207-16.
13. Vanneste JL, Cornish DA, Yu J, Voyle MD. P10c: a new biological control agent for control of fire blight which can be sprayed or distributed using honey bees. Acta Horticulture. 2002;590:231–6.
14. Kearns LP, Hale CN. Incidence of bacteria inhibitory to *Erwinia amylovora* from blossoms in New Zealand apple orchards. Plant Pathology 1995;44(5):918-24.
15. Mesarich CH, Rees-George J, Gardner PP, Ghomi FA, Gerth ML, Andersen MT, et al. Transposon insertion libraries for the characterization of mutants from the kiwifruit pathogen *Pseudomonas syringae pv. actinidiae*. PLoS One. 2017;12(3):e0172790.

16. Gristwood T, McNeil MB, Clulow JS, Salmond GP, Fineran PC. PigS and PigP regulate prodigiosin biosynthesis in *Serratia* via differential control of divergent operons, which include predicted transporters of sulfur-containing molecules. J Bacteriol. 2011;193(5):1076-85.
17. Moraru C, Varsani A, Kropinski AM. VIRIDIC-A Novel Tool to Calculate the Intergenomic Similarities of Prokaryote-Infecting Viruses. Viruses. 2020;12(11).
